# Neurorobots as a Means Toward Neuroethology and Explainable AI

**DOI:** 10.3389/fnbot.2020.570308

**Published:** 2020-10-19

**Authors:** Kexin Chen, Tiffany Hwu, Hirak J. Kashyap, Jeffrey L. Krichmar, Kenneth Stewart, Jinwei Xing, Xinyun Zou

**Affiliations:** ^1^Department of Cognitive Sciences, University of California, Irvine, Irvine, CA, United States; ^2^HRL Laboratories (formerly Hughes Research Laboratory), LLC, Malibu, CA, United States; ^3^Department of Computer Science, University of California, Irvine, Irvine, CA, United States

**Keywords:** neurorobotics, neuroethology, explainable artificial intelligence, interpretability, embodiment

## Abstract

Understanding why deep neural networks and machine learning algorithms act as they do is a difficult endeavor. Neuroscientists are faced with similar problems. One way biologists address this issue is by closely observing behavior while recording neurons or manipulating brain circuits. This has been called neuroethology. In a similar way, neurorobotics can be used to explain how neural network activity leads to behavior. In real world settings, neurorobots have been shown to perform behaviors analogous to animals. Moreover, a neuroroboticist has total control over the network, and by analyzing different neural groups or studying the effect of network perturbations (e.g., simulated lesions), they may be able to explain how the robot's behavior arises from artificial brain activity. In this paper, we review neurorobot experiments by focusing on how the robot's behavior leads to a qualitative and quantitative explanation of neural activity, and vice versa, that is, how neural activity leads to behavior. We suggest that using neurorobots as a form of computational neuroethology can be a powerful methodology for understanding neuroscience, as well as for artificial intelligence and machine learning.

## 1. Introduction

Neuroethological studies measure an animal's behavior under natural conditions rather than under artificial or limiting conditions that lead to erroneous conclusions about what the nervous system is responding to and how neuronal activity results in action (Ingle and Crews, 1985). Niko Tinbergen raised four questions for explaining behavior (Tinbergen, 1963): (1) *Causation*. What is the causal basis of the behavior? (2) *Ontogeny*. How does the behavior develop over the organism's lifetime? (3) *Adaptation*. How does an animal adapt to its environment? (4) *Phylogeny*. How did the behavior evolve over many generations? By focusing on the rich behavior, neuroethologists observe how this behavior “push[es] the envelope of what brains are capable of doing” (Dickinson and Moss, 2012). Recently, there has been a “call to arms” for a computational neuroethology (Datta et al., 2019). Neuroscientists now have amazing tools to probe the brain, including sophisticated anatomical tracers to identify pathways, optogenetics to manipulate neural circuits, and recording arrays that can monitor large populations of neurons during awake behavior. New techniques, such as those that automate measuring behavior, and virtual reality, that makes the laboratory setting appear more natural to the animal, allow researchers to examine these naturalistic behaviors while having the control over measurements of brain and behavior. In addition, the advent of deep neural networks and machine learning allows these large datasets to be analyzed in ways that could never occur before.

Still, with all these advances in neuroscience, we have difficulty measuring behavior while examining the whole brain at a resolution fine enough to understand how neural activity gives rise to behavior. It is ironic in a way that neuroscientists are turning to Artificial Intelligence (AI) methods to explain their data. For instance, using deep convolutional neural networks (CNNs) as models of hierarchical feature representation in the brain (Güçlü and van Gerven, 2015; Cichy et al., 2016; Yamins and DiCarlo, 2016), when AI has its own explainability issues. Others proposed similar models to synthesize control images to maximally activate specific neuron sites in the monkey IT cortex. However, often the synthesized images are not explainable and do not provide meaningful information about what the neurons are responding to (Bashivan et al., 2019; Ponce et al., 2019). Besides CNNs, Grossberg (2020) introduced how Adaptive Resonance Theory (ART) could implement a production system that incrementally learns without catastrophic forgetting and provides intrinsic explainability for its IF-THEN rule-based algorithms. Although explainability has emerged as a major research challenge in many domains of AI, in this paper we will focus on explainability of neural computations linked to observable robot behavior, as a unique paradigm of explainable AI.

For many years, situated models have been used to explain natural behavior. For example, the field of Artificial life (Alife) creates simulations and produces physical systems to show and study life-like processes (Bedau, 2003). Alife is very broad and ranges from cellular automata to evolving creatures (Sims, 1994). In general, Alife does not necessarily require an artificial brain or the embodiment of their agents. In an influential paper, “The brain has a body,” Chiel and Beer argued that jointly modeling neural control and the body morphology is a promising methodology for understanding adaptive behavior (Chiel and Beer, 1997). They called this computational neuroethology.

In this review, we follow this notion of synthetic methodology by looking at examples where robots with body structure are under neural control. Although many of the examples here may not be under the same natural conditions that are prevalent in neuroethology, we suggest that observing robot behavior in an environment while simultaneously analyzing the neural control is a powerful tool for understanding neuroscience, as well as machine learning.

Specifically, we examine how neurorobotics can explain AI and can be used as a form of “computational neuroethology,” and how this method can be used to explain AI. Neurorobots are robots whose control has been modeled after some aspect of the brain. Since the brain is so closely coupled to the body and situated in the environment, neurorobots can be a powerful tool for studying neural function in a holistic fashion (Krichmar, 2018). In a neurorobot experiment, the robot operates in the real world. It takes noisy sensory information from its environment and integrates this into actions. While this behavior is occurring, the neurorobotic researcher has the ability to examine the complete brain–that is, every neuron and synaptic change. Similar to a neuroethologist, but with far more control, the neuroroboticist can explain how these artificial brains give rise to behavior.

Neurorobotics has a long history of explaining how neural activity can lead to interesting, lifelike behaviors. In the late 1930's, the experimental psychologist, E.C. Tolman, who suggested that animals ranging from rats to humans have cognitive maps (Tolman, 1948), created a hypothetical schematic sow-bug to demonstrate the phenomenon of vicarious trial and error (Tolman, 1939). In the 1940's, Gray Walter built artificial “Tortoises,” which had rudimentary light sensors and collision detectors controlled by a simple analog circuit (Holland, 2003). A photoelectric sensor caused the steering mechanism to move the tortoise toward a light source. If the shell hit an obstacle, contact was made with a switch, causing the tortoise to back and turn away from the obstacle. When the tortoise's batteries were low, the tortoise went to its charging “hutch,” which was signaled by a light source. The important lesson from Gray Walter's tortoises is that interaction with the environment, even if controlled by a simple analog neural circuit, can result in realistic, natural looking behavior. A few decades later, neuroanatomist Valentino Braitenberg described a series of thought experiments in a short book titled “Vehicles” (Braitenberg, 1986). Each chapter of his book introduced a simple robot or vehicle that was a lesson in neuroscience. For example, by connecting the left light sensor to the right motor of these imaginary robots, and vice versa, Braitenberg described the difference between contralateral and ipsilateral connections and their effect on behavior. By changing the sign of the connection from positive to negative, he demonstrated the role inhibition plays in driving behavior. With these simple thought experiments, he introduced neuroscience concepts of sensorimotor loops, inhibition, and valence, as well as inspired generations of behavior-based roboticists.

Following these early attempts at explaining behaviors through simple neural circuits, the field of computational neuroethology started to emerge, which aims at producing animal-like behaviors through computational modeling of the brain (Beer and Chiel, 2008). Acknowledging that human beings are too complex to model as artificial intelligent agents, Randall Beer and his colleagues suggested that the design of AI systems should draw inspirations from simple natural animals, such as insects (Beer et al., 1990). They developed an artificial insect whose behavior was controlled by an artificial nervous system. With varying single neuron activity, the simulated insect demonstrated locomotion with different statically stable gaits mimicking natural insects. A lesion study on the locomotion controller revealed that higher and lower speed gaits were generated differently, with the former generated centrally and the latter more dependent on sensory information. This simulated insect was also able to perform wandering, edge-following, and feeding. As an early work in the field, Beer's simulated insect showed how artificial intelligence could be realized through modeling the neural circuitry of natural animals and embedding it in an environment where certain behaviors are required for the survival of the agent. Analysis of the model using a lesion method also deepened our understanding of how the neurocontroller generated complex dynamics. Instead of explicitly designing the neurocontroller of a robot, Nolfi and Floreano (2002) adopted an approach known as evolutionary robotics, with which they would evolve the neural network controller in an embodied and situated robot that was free to act in the environment. This approach allowed them to co-evolve the body and the brain, and to probe into the neural basis of sensory-motor coordination.

**Figure 1:**
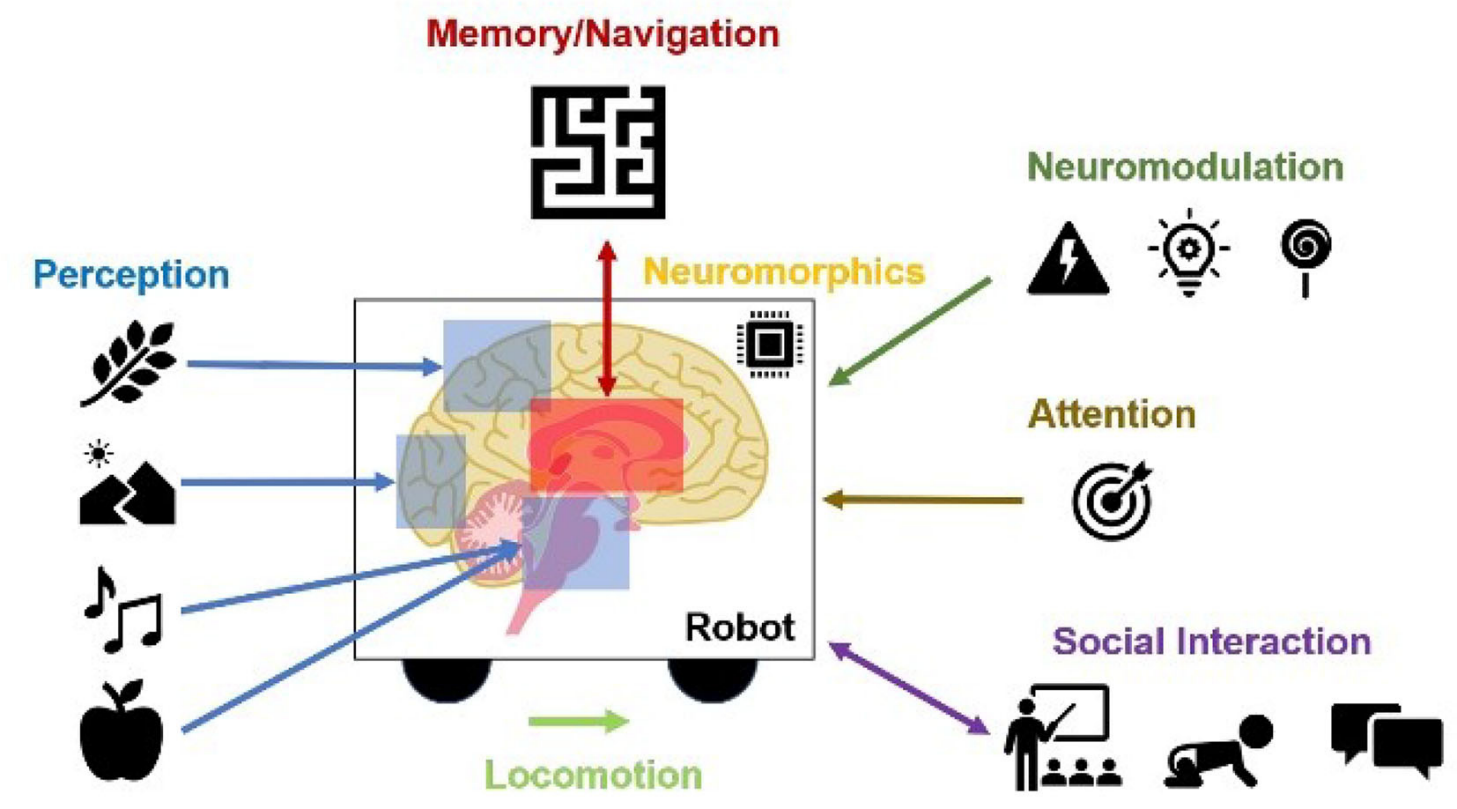
Contributions of Neurorobots as a Means toward Neuroethology and Explainable AI. The combination of neurorobotics and neuroethology enables explainable models tested in rich, embodied environments. Models of perception show how environmental stimuli are processed through systems of perception. From these perceptual experiences, memories and cognitive maps are built and tested. Interacting with the rest of the world, studies of locomotion, social interaction, and imitation learning show how agents engage with physical objects and other agents. Neuromodulation studies the processing of unexpected events, reward, and risk. Attention refers to visual search based on bottom-up scene-dependent stimuli and/or top-down goal-relevant inputs. Neuromorphic implementations learn from the power efficiency of the brain for benefits in hardware design and systems neuroscience.

In the remainder of this paper, we promote the idea of using neurorobotics as a means toward computational neuroethology. We discuss examples where neurally controlled robots are used to explain neural correlates of perception, memory, spatial navigation, neuromodulation, attention, locomotion, neuromorphics, and social interaction (Figure 1).

## 2. Perception

Because perception is closely coupled with action, embodied models can lead to better understanding of how perception leads to behavior (Ferretti and Chinellato, 2019). Transformations on the sensory input are done in the brain to yield an appropriate behavioral response to its environment. Analyzing these sensorimotor transformations to find the mechanisms through which the sensory input triggers the behavioral response is a major challenge in neuroscience (Kamali Sarvestani et al., 2013). Neurorobotics studies can help to explain these sensorimotor behavior responses and validate hypotheses surrounding their complex neural mechanisms.

### 2.1. Visual Perception

Sensorimotor skill development in infants involves learning neural representations of visual input and their associations to motor control mechanisms (Rutkowska, 1994). Of those, visuomotor skill computations can be understood using neurorobotics studies with appropriate biophysical constraints (Priamikov et al., 2016). For example, Klimmasch et al. (2017) simulated a binocular vision system with detailed human occulomotor biomechanics comprising six extraocular muscles to understand the development of self-calibration through active vision. In this study, sparse representations of input visual state and accurate vergence, the coordination of both eyes to maintain binocular vision, were simultaneously learned to control fixation on a timescale consistent with human infants. Efficient coding facilitated the emerged behavior, as the metabolic cost of muscle movement was minimized using reinforcement learning. This framework was extended to simultaneously also learn saccadic eye movements, which drive gaze to interesting regions in the scene (Zhu et al., 2017). Their work suggests that saliency-driven saccades guide the development of vergence control during early development and reinforces the findings that saccade-vergence interactions are learned through experience during childhood (Yang et al., 2002). This framework of joint development of sensory processing and eye movement control, termed as Active Efficient Coding (AEC), overall optimizes for coding efficiency of the sensory system and was proposed as a general framework of sensorimotor development.

AEC was also used to learn smooth pursuit eye movement control to track target objects using the iCub robot (Beira et al., 2006; Teulière et al., 2015). Using a general optimization framework to maximize the coding efficiency for visual input, a reinforcement learner developed the control policies for eye velocity even without being explicitly tasked to perform smooth pursuit eye movements. This learning mechanism was also used to develop iCub's motor skills for both smooth pursuit and vergence eye movements in presence of a stimulus moving in 3D (Lelais et al., 2019). While doing so, the AEC framework learns the entangled basis set of disparity and motion that is sparsely activated. Their results suggest that the AEC framework continuously improves tracking and vergence performance until the physical constraints are reached, such as camera and motor resolution or the capacity of the basis dictionary. Further, the basis functions learned by AEC show a mix of independent and joint tuning for disparity and motion, which is comparable to the population tuning profile in the medial temporal (MT) region of the primate brain (Smolyanskaya et al., 2013; Czuba et al., 2014). These results suggest that both vergence and pursuit eye movement develop from the same objective of maximizing the coding efficiency of the visual system. This is consistent with many physiological findings that efficient coding is a ubiquitous encoding strategy used by diverse organisms across modalities, see (Beyeler et al., 2019) for a detailed review.

An important sensorimotor cue for visual perception is motion parallax, which complements binocular disparity in perception of depth. Motion parallax is the displacement in the retinal position of the projection of an object as the observer moves through the environment. Biological depth estimation systems utilize parallax resulting from subtle movements, such head/eye rotations for fixation. For example, before striking a prey, many birds and insects generate parallax though eye/head movement to obtain a perceptual judgment of distance. The active scanning behavior can be naturally studied using a robotic implementation to explain its association with depth perception. Kuang et al. (2012) replicated this behavior on a humanoid robot and observed that the parallax-based distance estimates emerge during compensatory head/eye movements to fixate on a target during self-motion. When the robot rotated its head at a constant speed, its eyes rotated in the opposite direction to position the fixation object at the center of the image. During these eye movements, objects at different depths and eccentricities translated by different amounts on the projected image. By comparing projected object locations between two time points and by factoring out eccentricities, the depth of the objects were recovered. Their robotic demonstration of parallax showed that vision systems should incorporate temporal changes in the environment due to the agent's own behavior to identify hidden structures in the scene, which is not explored by most machine vision systems, but is prevalent in biological embodied vision systems.

Behaviors such as locating prey or recognizing and escaping predators require rapid visuomotor responses. In one study, the approach and escape behavior of the lamprey eel was investigated with a neurorobotic approach (Youssef et al., 2020). This study validated a biologically inspired visuomotor controller from Kamali Sarvestani et al. (2013) and pioneered the use of event-driven cameras in an underwater robot. Visual input from the cameras stimulated the visuomotor controller. The visuomotor controller evoked multiple behaviors that the robot would use to respond to attractive and repulsive visual cues as it navigated through its environment. The visuomotor controller was implemented as a neural network comprised of three subsystems: (1) a stimulus prioritization strategy and position encoding subsystem modeled after the optic tectum in vertebrates; (2) a behavior arbitration subsystem modeled after the basal ganglia that allows the controller to designate a single response when faced with competing stimuli; and (3) a network output generating the robot's Central Pattern Generator (CPG) for speed and turning locomotion.

The robot's behavior was determined by the output of the behavior arbitration network. Each behavior out of approaching, escaping, and avoiding, had an associated subthalamic nucleus (STN), external globus pallidus (GPe), and internal globus pallidus (GPi) neuron. The outputs of the behavior arbitration sub-network are the inhibitory connections between each GPi neuron and all response layer neurons of the same behavior. Four behavioral studies conducted to examine the performance of the behavior arbitration sub-network. Behaviors were produced in response to color cues. The robot was placed in a pool and faced with a single stationary attractive stimuli, a stationary repulsive stimuli, two stationary attractive stimuli, or two stationary stimuli, one attractive and one repulsive. These neurorobotic experiments demonstrated how behavior arbitration networks, which mimic the basal ganglia, have the ability to rapidly choose the desired behavior based on visual sensory information.

### 2.2. Tactile Perception

**Figure 2:**
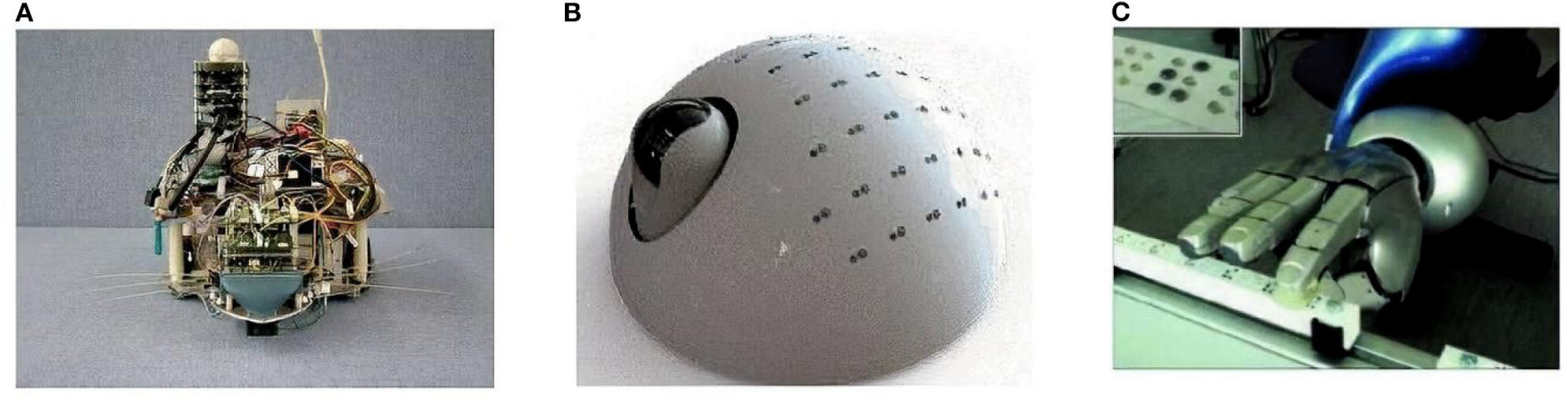
Examples of neurorobots using tactile perception of the environment to generate explainable behavior. **(A)** The Whiskerbot, a robot that emulates rodent whisker sensory system for navigation (Pearson et al., 2007). **(B)** CARL-SJR, an anteater robot that interacts with human users by flashing multi-colored LEDs in response to rubbing gestures on its back (Bucci et al., 2014). **(C)** A neurorobotic hand for fine tactile sensing, such as reading Braille characters (Bologna et al., 2013).

The neurorobotic approach has been used to explain how sensorimotor integration in the brain and the peripheral nervous system can result in tactile exploration of the environment. These studies range from active whisking to manipulation with robotic hands. In particular, tactile perception uses clever and ingenious use of materials to facilitate the compliance and resolution necessary for these tasks (see Figure 2).

**Figure 3:**
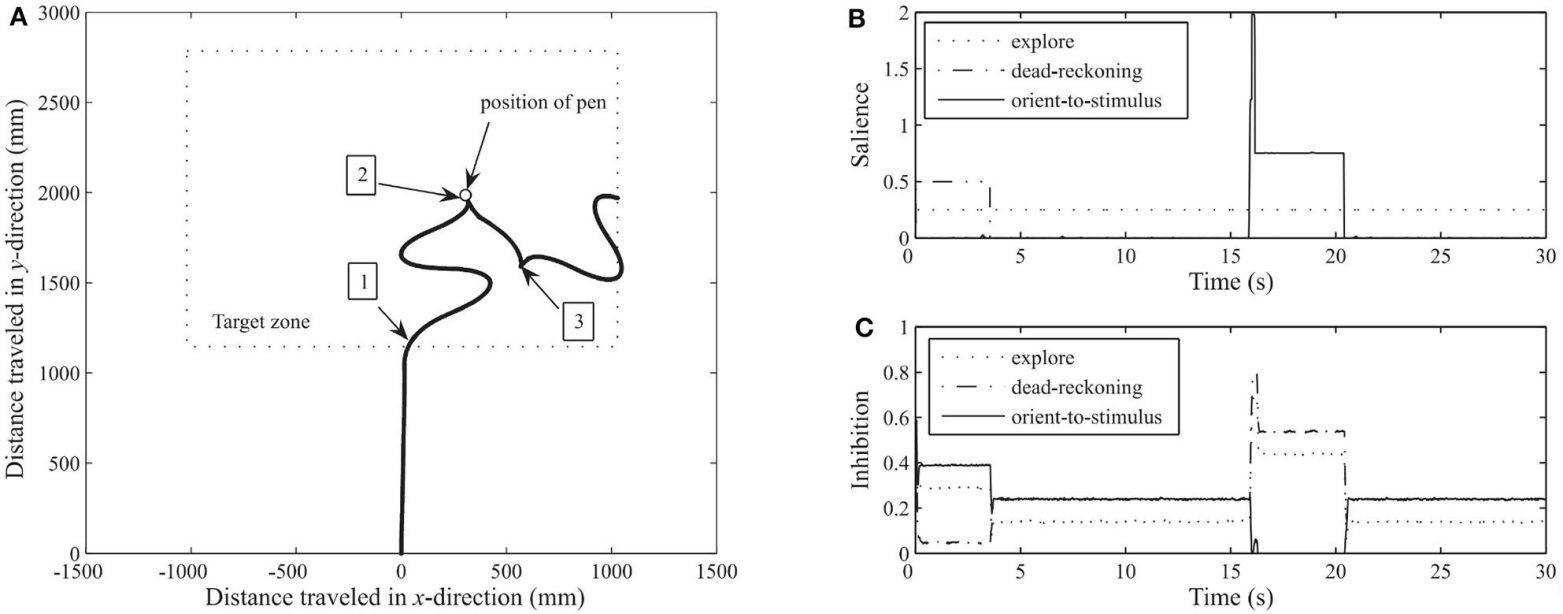
Thirty-second run of the Whiskerbot. **(A)** Progress of robot as it moves across the floor, taken from monitoring the rotation of each wheel. 1: Robot reaches target zone using the dead-reckoning behavior at which point it switches to exploring. 2: Contact is made by one of the whiskers with a pen and an orientation response is enacted. 3: After orientation and fixed reverse maneuver the robot continues to explore. **(B)** The salience from each behavior throughout the run. Dead-reckoning begins as the most salient behavior but changes to explore upon reaching the target zone, and then at about time 16 the orient-to-stimulus behavior spikes in response to contacting the target before returning to exploratory behavior. **(C)** The resultant level of inhibition projecting back from the basal ganglia to each behavior. When the saliency of a behavior increases the inhibition projected back to the basal ganglia decreases. Figure and caption are reproduced and adapted from Pearson et al. (2007).

The Whiskerbot is a biologically-inspired robotic implementation of the rodent whisker sensory system that drives sensorimotor behaviors using tactile perception to navigate the environment and orient the snout toward detected objects (Pearson et al., 2007). The Whiskerbot uses artificial whiskers and a neural network architecture modeled after the rodent central nervous system,which is responsible for whisker sensory processing (see Figure 2A). The whiskers are moved back and forth in a regular sweeping pattern called whisking to extract spatial and textural information from the environment. Similar to gaze orientation in the primate, but within the tactile domain, the superior colliculus in rodents has strong input from their vibrissae causing head orienting behavior toward detected stimulus. The functionality of the superior colliculus is implemented in the Whiskerbot's neural network along with a network modeled after the basal ganglia that decides which of three actions the Whiskerbot should perform depending on salience and sensory input, disinhibiting the necessary motor projections. Using its whiskers, the Whiskerbot can orient to a tactile stimulus, navigate through dead reckoning, and explore the environment with a sinusoidal searching pattern. Figure 3 shows one of these trials in which all of these behavioral responses are performed by the network, showing the network behavior over the duration of the trial. The behavior of the Whiskerbot during the trial and the recorded neural network activity demonstrate the correct functioning of the network and artificial whiskers. The study shows that embodying biologically inspired sensors and networks in a neurorobot enables one to study realistic animal behaviors in a real world setting.

Bucci et al. (2014) proposed an interactive tactile neurorobot to explore sensory decoding by a spiking neural network (SNN) model of the somatosensory cortex (see Figure 2B). To support tactile interaction with people, they developed a robotic platform, named CARL-SJR, which had an array of trackballs on the surface of a hemispherical shell to signal the direction and velocity of tactile stimuli. Moreover, the robot communicated by flashing multiple colored LEDs in response to tactile stimuli. The SNN learned different spatiotemporal hand movement patterns across the trackball array using biologically plausible synaptic updates. Their results showed that temporal decoding of neuron population activity accurately predicted the interactive tactile input in real time. With its unique design, CARL-SJR provides a potent platform for studying neural encoding of touch and human-robot interaction.

Bologna et al. (2013) proposed a closed-loop neurorobotic system to perform fine-grained touch recognition through active sensing (see Figure 2C). The closed-loop system consists of: (1) an artificial touch sensor providing an array of analog responses to tactile stimulation; (2) a network of primary neurons that convert the analog touch input into spiking activity; (3) a network of secondary neurons that further processes primary afferent signals for downstream motor control and recognition; (4) a probabilistic classifier for tactile input recognition; (5) high and low-level motor controllers for fine motor movement to facilitate active sensing-based optimal classification; and (6) a robotic arm-hand setup. They proposed mechanoreceptors and cuneate neurons in the brainstem as the neural correlates of the primary and secondary model neurons, respectively, which transform input for downstream cortical processing. When tested on Braille characters, their system achieved approximately 95% discrimination accuracy after only 350 ms of stimulus onset. Moreover, the resulting fingertip kinematics were consistent with human Braille readers. Their results suggest that fingertip kinematics can be adapted online for fine-grained tactile sensing in a closed-loop active sensing procedure to maximize extracted information for recognition.

In another study of fine-grained touch recognition through compliant robotic manipulation, a recent study developed a neuro-inspired architecture to demonstrate dynamic touch tasks (Rongala et al., 2019). They embodied a spike-based neuromorphic encoding of tactile stimuli to emulate the discrimination properties of cuneate nucleus neurons based on pathways with differential delay lines. Specifically, the robot arm and wrist were able to discriminate edge orientations with high fidelity. The study showed how the principle of differential delay matching led to encoding of the stimulus orientation.

### 2.3. Auditory Perception

The ability to perceive sound is important for many tasks, such as navigation and localization. An example of auditory sensorimotor integration that can be explained through neurorobotics is the owl's ability to locate prey. The localization system in the barn owl uses the time difference of sound arrival between ears to calculate the azimuth and the amplitude difference of sound between the ears to calculate elevation (Konishi, 1993). However, the mechanism that processes these cues for localization must adapt to individual differences in head/ear sizes and in particular, for young barn owls who are able to adapt to drastic changes of their sensory conditions. To study this adaptation behavior, Rucci et al. (1999) developed a detailed computational model of spatial localization in the barn owl to control orienting behavior in a robotic system in the presence of visual and auditory stimuli. Their setup included a robotic head with a camera and two lateral microphones, which was controlled by a computational model of the neural pathways involved in the localization process. The adaptation behavior was achieved via plasticity of the synapses based on the activity of a diffusing modulatory system that signaled the occurrence of visually significant events, such as the positioning of the target on the fovea. Similar experience-dependent changes were also observed in physiological studies (Brainard and Knudsen, 1993). This study showed that adaptation of behavior does not require new neural structures and can be implemented via experience-dependent plasticity of existing synaptic connections, in this case by integrating visual modulation with auditory processing.

Auditory cues are also used by insects for sound localization or phonotaxis. Barbara Webb created a neurorobotic model of cricket phonotaxis to better understand how the cricket can locate a mating call (Webb and Scutt, 2000). They developed a simple spiking model consisting of only four neurons to show a surprisingly complex sound localization. By comparing the latencies of sound arrival between the vibration sensors on the legs of the cricket, the source of a sound could be localized and tied to the motor outputs controlling heading direction. The distance between the legs and the material of the head was tightly tuned to the frequency of a cricket song and critical for localization performance. Moreover, the models are able to capture specific qualities of the sound, such as frequency and repetition, which the crickets use to find other crickets. This neurorobotic experiment showed how the tight coupling between neural processing and morphology can lead to successful behavior.

## 3. Memory and Navigation

Spatial representations and declarative memory are necessary for navigation and exploration, inspiring many neurorobotics applications (Zeno et al., 2016). As we will see in this section, neurorobotics demonstrations of navigation help to explain how the brain builds cognitive maps of their environments and uses these maps to plan actions and achieve desired goals. The cognitive maps themselves are built over time, through repeated experiences of exploring the environment. Memory models explain how these singular experiences consolidate into summary representations of space. Furthermore, memory models allow robots to explain their actions. For instance, a robot might be able to recall past episodes of its experiences with handling an object or visiting a location to explain its current actions. Robot experiments allow us to test such models that link perception, memory, cognitive mapping, and spatial navigation (Arbib, 2020).

### 3.1. Spatial Navigation

#### 3.1.1. Insect Navigation

Insects often perform random foraging when searching for food resources, but are able to go back straight to the nest using landmarks. This requires path integration, which is the ability to keep track of where oneself is in relation to some starting location, and is an important function for foraging animals. Menzel and Greggers (2015) suggested that insects, such as honeybees, use a mental map to guide navigation. This map contains action memories that store the spatial/temporal relations of landmarks, and also assigns “meanings” to these landmarks.

To investigate how insects perform path integration, Lambrinos et al. (2000) used a robotic agent to model the navigation ability of desert ants. The robot was equipped with polarization vision and with a panoramic visual system that was functionally similar to the insect eye. The robot demonstrated stable path integration ability through the accurate estimate of the robot's heading with respect to the sun. The robot also used a snapshot model for visual landmark navigation, which allowed the robot to store snapshot images of landmarks and use them to compare with its current retinal image while returning to the home location. This study provided realistic implementations of the two navigation methods employed by insects: path integration for long-distance traveling, and visual landmark navigation when the target location is within a shorter distance.

**Figure 4:**
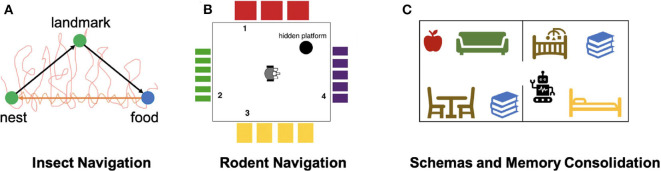
Models of Navigation and Memory. **(A)** Neurorobotic experiment on insect navigation (Mathews et al., 2009). The SyntheticAnt robot wanders an arena with its trajectory marked in red, searching for a food source using chemical sensing. After arriving at the food location, the robot is able to use the encountered visual landmark to compute a trajectory back to the nest, as indicated by the orange arrow. The robot also has a memory of the landmark that it is able to travel from one location to the other without odor cues. **(B)** Neurorobotic experiment on place cell navigation (Krichmar et al., 2005). The Darwin X robot sees different visual patterns from each wall in the room, developing place fields that allow it to learn navigation-based tasks. **(C)** Neurorobotic model of schemas (Hwu et al., 2020). By learning schemas in the form of objects belonging to different rooms, the Toyota HSR robot can disambiguate task commands, such as using its current context to pick up a book.

In another study of insect navigation strategies, Mathews et al. (2009) implemented an insect-inspired navigation model using a ground robot to demonstrate the integration of landmark recognition, chemical search, and path integration (Figure 4A). Landmark recognition was implemented through a neural network that performs image recognition of visited landmarks. Path integration was acquired using head direction accumulators (HDA), which accumulated sensory information to encode the direction and distance to landmarks, and were reset after encountering landmarks. The model also maintained short-term and long-term memories to store visual cues and HDA information and used them to compute the optimal route to the goal landmark. The model, when implemented on a mobile robot, showed successful landmark navigation and homing behaviors, which were robust to landmark addition or removal. The robot also demonstrated a probabilistic use of memory, which supported generalization of homing behaviors. This study showed that goal-directed navigation can be achieved with ego-centric cues, which establishes the relationship between objects in the world and the observer itself, and without an allocentric map-like representation of the environment, which reflects an object-to-object relationship.

In general, these and other neurorobotic studies of insects demonstrate how different navigation strategies can be supported by simple neural networks. Evidence suggests that vertebrates may be using more sophisticated memory structures to navigate, but they may also be utilizing some of the homologous strategies that have been analyzed in insects (Collett and Collett, 2002).

#### 3.1.2. Rodent Navigation

Goal-directed navigational behaviors have been extensively studied in mammals, especially in rodents. Rodents are suggested to maintain a “cognitive map” of the surrounding environment. Neurophysiological studies have revealed distinct representations for spatial elements such as place, distance, direction, and boundaries in the rodent brain. It is intriguing how these different representations emerge in different regions of the brain and how these regions coordinate to use these cognitive maps to guide navigational decisions. Neurorobots serve as great tools to implement neurobiologically inspired navigation systems, which on one hand, integrate navigation strategies employed by naturally intelligent agents, and on the other hand, provide a convenient platform to test how neural network activities give rise to various behaviors. In a review of recent advancement in neurobiologically inspired navigation systems for mobile robots, Zeno et al. (2016) suggested that such systems could be classified into three main types: place cell centric systems, theoretical cell centric systems, and grid cell centric systems.

Place cell centric systems are built on the idea that hippocampal neurons respond specifically to locations in the environment. Place cells have been found primarily in the CA1-CA3 regions of the hippocampus (HPC) (O'Keefe and Nadel, 1978). In one neurorobotic example, Arleo et al. (2001) showed that place cell activity could emerge from learning the correlation between visual cues represented with superficial lateral entorhinal cortex (sLEC) cell activity and path integration elements represented by head-direction signals in the superficial medial entorhinal cortex (sMEC). By using these place cells as basis functions for reinforcement learning, they also showed that the robot was able to perform goal-directed navigation. In another study, Krichmar et al. (2005) showed that place-specific units similar to place cells emerged through combining visual and self-movement cues during exploration (Figure 4B). With this brain-based robot, they were able to identify different functional hippocampal pathways and observe how these pathways influence place field activity and behaviors during navigation. Fleischer et al. (2007) used a later version of this mobile robot to demonstrate the emergence of journey-dependent place cell responses, some of which were retrospective, where neural activity is present after choice, and others were prospective, in which neural activity “predicts” future route selections. Through backtrace analyses on the network activity during the robotics experiment, Fleischer et al. (2007) also concluded that the hippocampus had a stronger influence on the journey-dependent cells, suggesting an important role of the hippocampus in remembering the past and predicting the future.

Neurorobotics has been used to predict the existence of response properties that could support effective navigational behaviors, but are not yet identified in neural recordings. Cuperlier et al. (2007) introduced “transition cells” which are sensory-motor units that explicitly code the spatiotemporal transitions between places. They implemented a neural network model containing both place cells and transition cells on an autonomous robot. Transition cells activity guided the choice of the movement to perform. They argued that transition cells provided important information for localizing the robot, and they also justified that transition cell activity would be hard to isolate from a place cell activity, which may explain why this cell is not yet discovered.

Some models have been designed to solve complex robotic tasks with a neurobiologically inspired approach. For example, Milford et al. (2004) introduced “pose cells” which are essential components of the RatSLAM system. The RatSLAM is a neural inspired Simultaneous Localization and Mapping (SLAM) system, in which an agent builds an internal map of the environment while keeping track of its current locations. RatSLAM was designed to approximate the navigational functions of the hippocampal complex with competitive attractor networks. The network formed pose cells, which conjunctively represent the beliefs about the location and orientation of the robot, and can be seen as a combination of grid cells and head-direction cells. The network performed path integration by taking in motor information and external visual cues. These visual cues were converted into activity of local view cells and formed associations with consistent pose cells. RatSLAM was tested on a real robot and demonstrated the ability to create consistent representations of the environment in an online incremental fashion with the fictitious pose cells.

Through navigation experiments with robots employing the RatSLAM system, Milford et al. (2010) suggested that conjunctive grid cells found in the dorsocaudal medial entorhinal cortex (dMEC) may play a role in reducing sensory uncertainty during path integration and landmark calibration. They showed that the cells in RatSLAM had similar characteristics to rodent grid cells in behavioral experiments, and the model cells can encode multiple hypotheses of spatial location and orientation. This representation of uncertainty allowed the robot to navigate in a perceptually ambiguous environment.

### 3.2. Schemas and Consolidation

Many of the brain areas involved in navigation are also involved in memory processes, such as the formation of schemas. A schema is defined as a collection of objects or concepts that belong to a shared context. For example, objects belonging in the same room are associated into the same schema since they are often seen together in the same context. Hwu and Krichmar (2019) presented a model of how multiple schemas are formed over time, allowing overlapping tasks to be performed without catastrophic forgetting. The model contains three components: a representation stream, an indexing stream, and a neuromodulatory area. The representation stream is responsible for learning representations necessary to perform a task, with sensory cues as input and actions as output. This area is sufficient for learning individual tasks. However, if a new task is trained, previously-learned tasks are forgotten as the representations are overwritten. This problem is avoided through use of the indexing stream, which encodes schema information through the medial prefrontal cortex (mPFC) and projects sparse patterns of activity to each level of the representation stream. The indexing occurs along the dorsal-ventral axis of the hippocampus (HPC) in a hierarchical fashion. The addition of a neuromodulatory area explored how schemas are used to detect novelty and familiarity. When novel information was presented within a familiar schema, the neuromodulatory area increased the learning speed of the information, consistent with theories of schema consistency and encoding (Van Kesteren et al., 2012). The model was applied to a neurorobotics demonstration to show how schemas are formed and used in real-world environments (Hwu et al., 2020). The Toyota Human Support Robot (HSR) used the schema model to encode schemas in two separate rooms (Yamamoto et al., 2019), learning to retrieve objects from both rooms (see Figure 4C). Despite the fact that some objects were present in both rooms, the robot retrieved the one consistent with its current schema, and did not get confused when switching between tasks separately trained in each of the schemas. The robot was able to rapidly learn how to retrieve novel objects within the existing schemas and switch between tasks without confusion. Furthermore, the encoding of schemas helped the robot retrieve items it had never explicitly been trained to retrieve. The model and the robotics experiments showed how schemas could be created and updated in a neurbiologically plausible architecture. In particular, the robotics experiments demonstrated how schemas might be applied in everyday activities.

## 4. Neuromodulation

Neuromodulatory systems are critical for vertebrates to respond to the environment appropriately and adjust to changes. Since these responses and adjustments are demonstrated as behaviors, neurorobotic experiments in which robot behaviors are observable and controllable are perfect options for researchers to study neuromodulatory systems.

**Figure 5:**
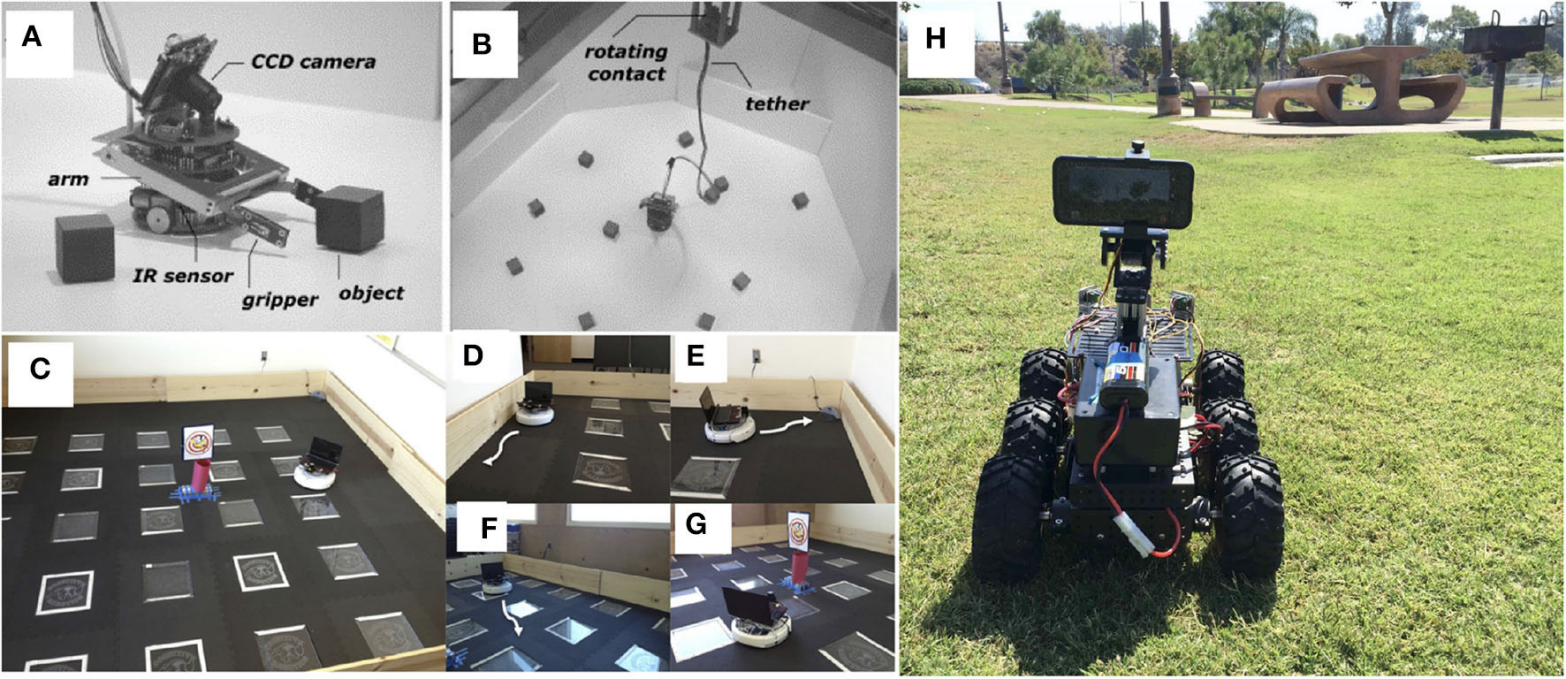
Examples of robots used for neuromodulation experiments. **(A,B)** Robot and environment to test dopaminergic value prediction (Sporns and Alexander, 2002). **(C–G)** Roomba robot used to test anxious and curious behavior (Krichmar, 2013). Behavioral primitives included wall follow **(D)**, hide in nest **(E)**, explore room **(F)**, and investigate object **(G)**. **(H)** Android-based robot used for serotonin patience experiments (Xing et al., 2019).

For instance, Krichmar (2013) designed neurorobotics experiments to test the hypothesis that high levels of serotonin (5-HT) lead to withdrawn behavior by suppressing dopiminergic (DA) activity and that high levels of DA or low levels of 5-HT lead to exploratory behavior (see Figures 5C–G). In this experiment, a robot responded to sensory events including detecting light, objects and bumps, each of which could trigger different neuromodulatory neurons, which in turn triggered different behavioral responses. When the robot was placed in a new environment, it demonstrated anxious behavior similar to rodent behavior, such as staying near its nest or following walls. When the robot or rodent became comfortable with this environment, it became curious and explored the area. The resulting behavior and neural activity in the model supported the hypothesis that top-down signals from the frontal cortex to the neuromodulatory areas were critical for handling both stressful and positive valence events.

In another neurorobotic study of neuromodulation, Sporns and Alexander (2002) studied the DA system but from the perspective that the value signal from the DA system could influence the magnitude and direction of synaptic plasticity (see Figures 5A,B). The value signal from the DA system is involved in synaptic learning via the value-dependent learning rule, and then the synaptic updates result in behavioral changes. This study supported the hypothesis that neuromodulators facilitate the induction and expression of long-term synaptic plasticity within our brain.

**Figure 6:**
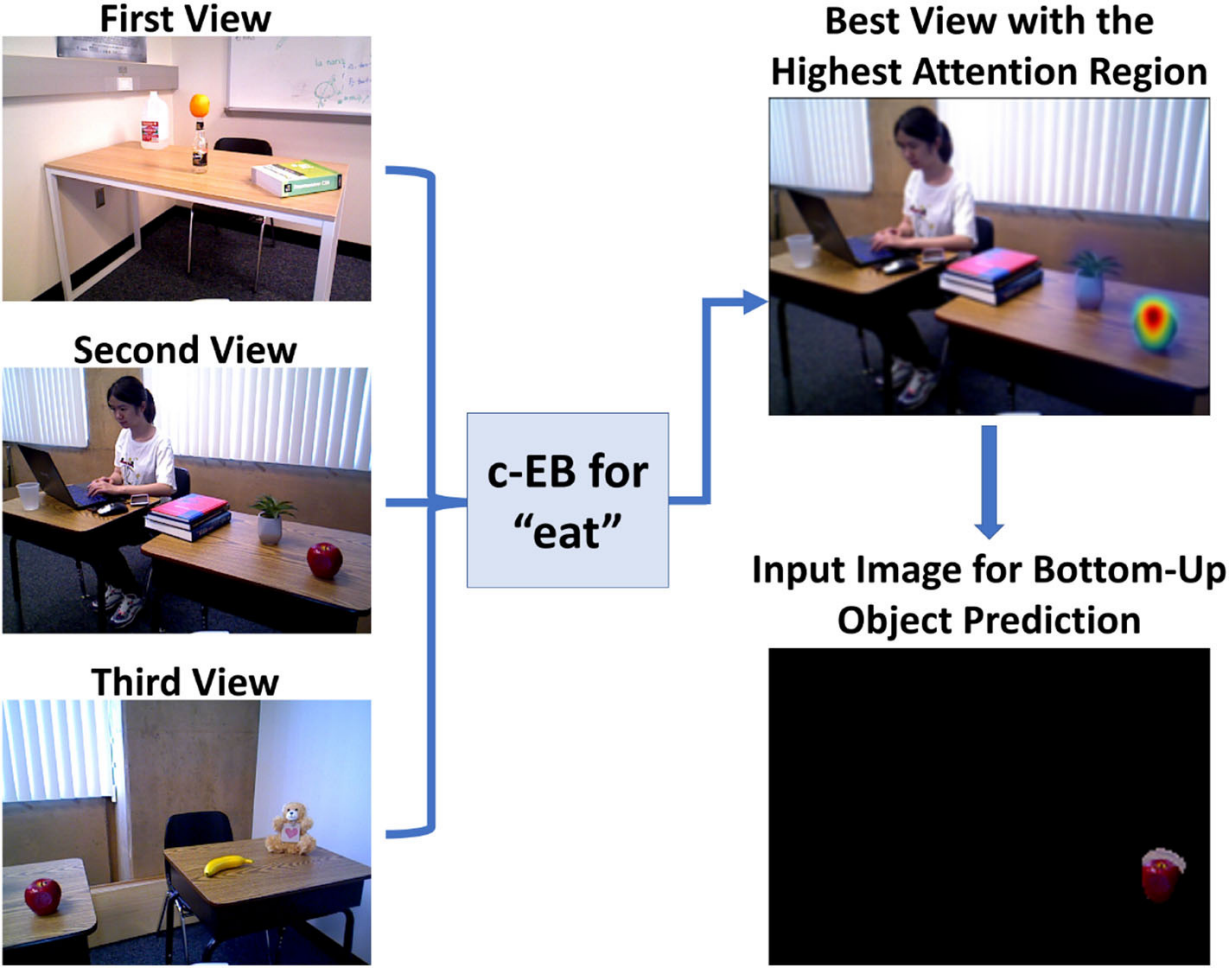
Toyota Human Support Robot implementation for the goal-driven perception model, including the top-down attentional search process for a guessed action “eat” based on three different real indoor views to select the highest attention region for bottom-up object prediction. Figure is reproduced from Zou et al. (2020).

The neuromodulatory system also regulates attention allocation and response to unexpected events. Using the Toyota HSR, the influence of the cholinergic (ACh) system (Sarter et al., 2005) and noradrenergic (NE) systems (Berridge and Waterhouse, 2003) on goal-directed perception was studied in an action-based attention task (Zou et al., 2020). In their experiment, a robot was required to attend to goal-related objects (the ACh system) and adjust to the change of goals in an uncertain domain (the NE system). Four different actions (i.e., “eat,” “work-on-computer,” “read,” and “say-hi”) were available in the experiment and each of them was associated with different images of objects. For example, the goal action “eat” might result in attention to objects such as “apple” or “banana” while the action “say-hi” should lead attention to a “person.” During the experiment, the goal action changed periodically and the robot needed to select the action and object it thought the user wanted based on prior experience. Their model demonstrated how neuromodulatory systems can facilitate rapid adaptation to change in uncertain environments. The goal-directed perception was realized through the allocation of the robot's attention to the desired action/object pair (see Figure 6). Section 5 provides more examples of how neuromodulated attention can affect behavior. The examples above demonstrated how neuromodulators control the attention for goal adaptation and perception.

**Figure 7:**
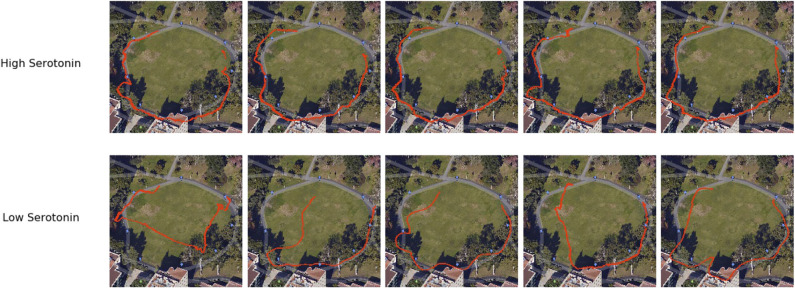
Examples of flexible navigation behaviors generated from different 5-HT levels (Xing et al., 2019). Red traces are drawn from GPS readings from the phone mounted on the robot. The robot searched for and found all waypoints with high 5-HT **(top)**, but skipped some waypoints and took shortcuts to other waypoints when 5-HT was low **(bottom)**.

5-HT activity is thought to be important for regulating anxious behavior and harm aversion. But recently, 5-HT has been shown to have an influence on patience control (Miyazaki et al., 2018). To test this idea in a real-world application, Xing et al. (2019) designed a robotic navigation experiment to show how changing the simulated 5-HT level could affect the amount of time the robot spent searching for a desired location. In their experiment, the robot searched for GPS waypoints in different outdoor environments (see Figure 5H). If the 5-HT level was low or a waypoint was difficult to find, the robot became impatient and searched for another waypoint. From this, flexible navigation strategies emerged in the observed robot behavior, such as calling off the search of a difficult to find landmark due to impatience or taking advantage of a smoother but longer route by being extra patient. Some examples of navigation traces are shown in Figure 7. This study provides an example of how 5-HT neuromodulators regulate patience levels in optimal control.

## 5. Attention

**Figure 8:**
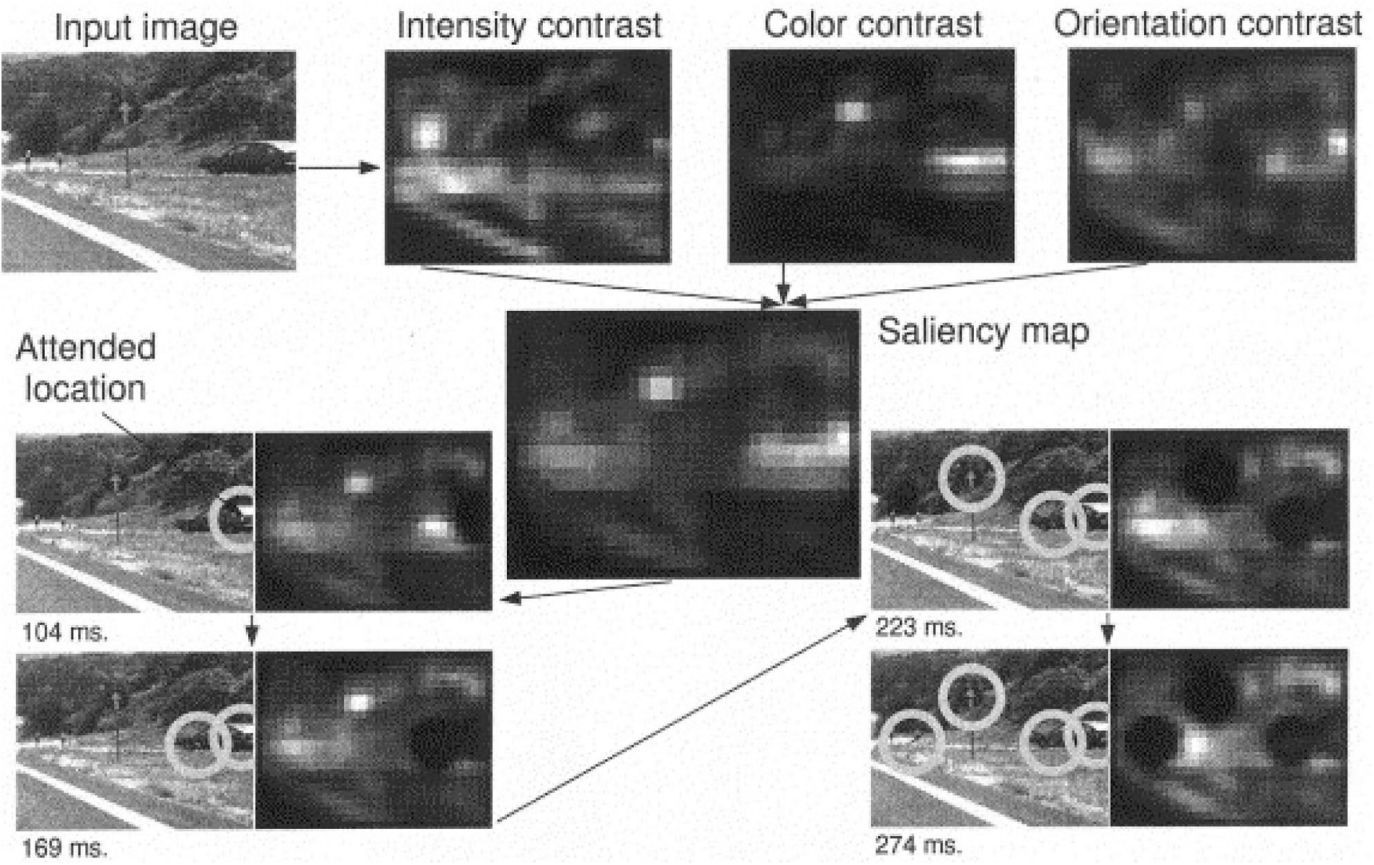
Example of the working of the saliency-based search model with a 512 × 384 pixels color image. Feature maps are extracted from the input image at several spatial scales, and are combined into three separate saliency maps (intensity, color, and orientation) at scale 4 (32 × 24 pixels). The three conspicuity maps that encode for saliency within these three domains are combined and fed into the single saliency map (also 32 × 24 pixels). A neural winner-take-all network then successively selects, in order of decreasing saliency, the attended locations. Once a location has been attended to for some brief interval, it is transiently suppressed in the saliency map by the inhibition of return mechanism (dark round areas). Note how the inhibited locations recover over time (e.g., the first attended location has regained some activity at 274 ms), due to the integrative properties of the saliency map. The radius of the focus of attention was 64 pixels. Figure and caption are reproduced from Itti and Koch (2000).

**Figure 9:**
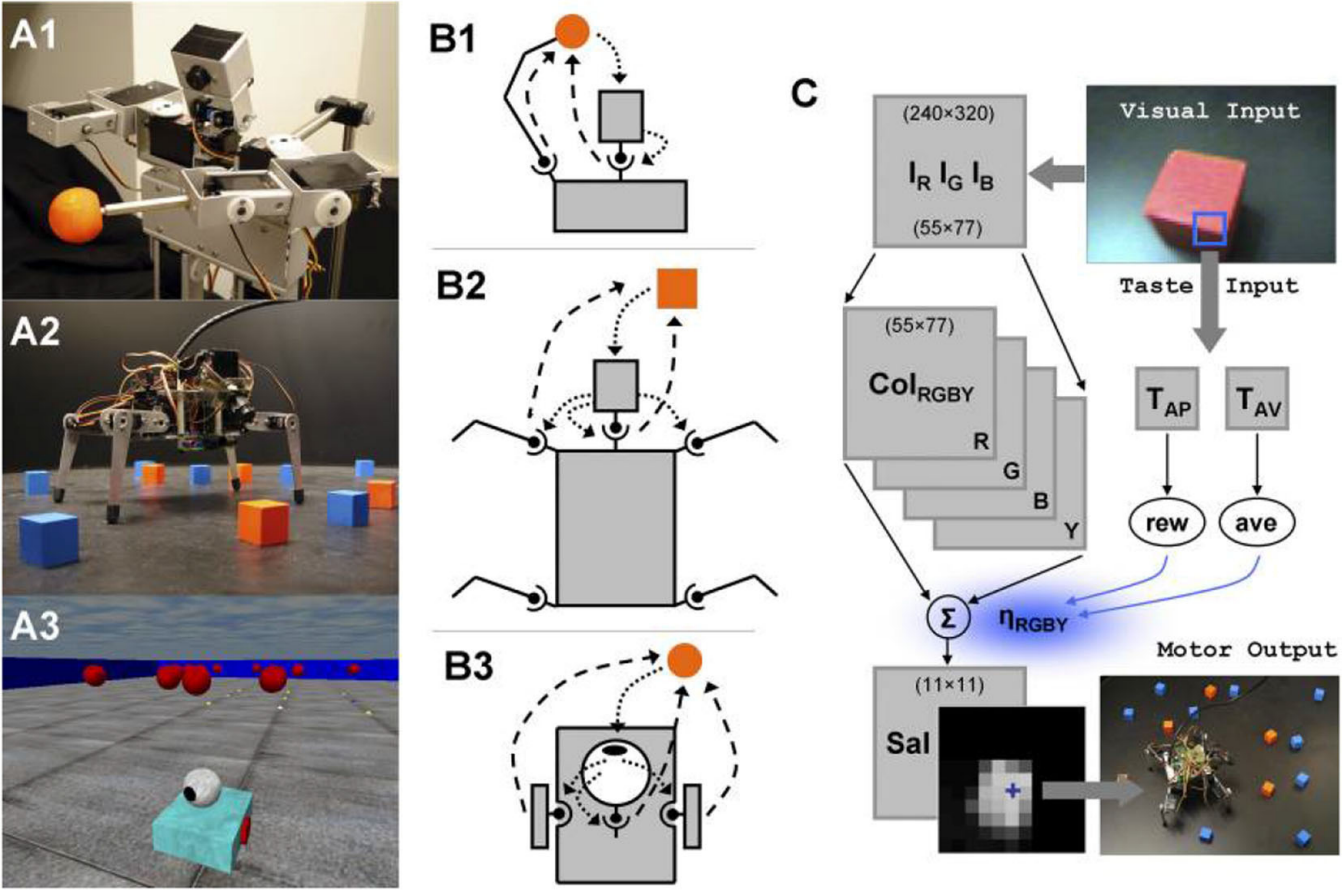
Robots, sensorimotor interactions, and the neural control architecture that adapts the saliency-based visual search model by using three morphologically different robotic platforms, including **(A1)** a humanoid robot called *Roboto*, **(A2)** a mobile quadruped called *Strider*, and **(A3)** a mobile wheeled robot called *Madame* (Itti and Koch, 2000; Lungarella and Sporns, 2006). **(B1)** Roboto engages in sensorimotor interactions via the head system and arm movements; sensory → motor (dotted arrows), motor → sensory (dashed arrows). **(B2)** Strider engages in sensorimotor interactions via the head system, as well as via steering signals generated by the head and transmitted to the four legs. **(B3)** Madame's behavior consists of a series of approaches to colored objects and ovations. Fixations to the objects are maintained by independent action of head and body. **(C)** Neural control architecture. The components common to all robots are color image arrays, color-intensity map, and saliency map. The peak of the saliency map (blue cross) determines the pan-tilt camera motion and body steering. The neural system contains a value system with taste sensory inputs relayed via a virtual taste sensor (blue square in visual image) to taste neurons, which in turn generates reward and aversiveness signals used to modulate the strengths of the saliency factors. Figure and caption are reproduced and adapted from Lungarella and Sporns (2006).

Models of attention have been proposed to explain mechanisms for efficient visual search in humans and artificial systems, under both bottom-up (scene-dependent) and top-down (task-driven) control (Tsotsos et al., 2015; Tanner and Itti, 2017, 2019). The saliency-based search model proposed by Itti and Koch (2000) represents a pure dependence on the bottom-up saliency. Instead of requiring any goal in the top-down direction to shift attention, the network highlights important feature locations in the order of decreasing saliency. The saliency map is a combination of three separate feature maps (intensity, color and orientation) that encode saliency within these image features. As long as the intended context has not been found, the network suppresses the visited locations and keeps searching for the next location with the highest saliency in the map (see Figure 8). Figure 9 shows several neurorobotic applications of this saliency-based visual search model. In these examples, the robot orients toward the peak location of the saliency map by moving its pan-tilt camera motion or steering its body. Such neurorobotic behavior closely matches saccadic eye movements during stimulus-driven attention (Hoffman and Subramaniam, 1995; Liversedge and Findlay, 2000). The model showed how the bottom-up salient stimuli could drive visual search to attend to locations quite different from their surround. The robotics experiments further emphasized how a saliency-based visual system could effectively direct the information flow between sensory, neural, and motor variables.

A more biologically realistic attentional mechanism would not only include bottom-up stimulus-driven signals, but also a top-down task-driven path to effectively direct attention to goal-relevant inputs (Baluch and Itti, 2011). These goals can be unknown initially and thus require learning through experience. Furthermore, they can shift without warning. Goal-driven perception helps filter out less relevant stimuli and instead focus on critical stimuli which require an immediate response. This procedure can be observed in the cholinergic system in the brain (Baxter and Chiba, 1999; Oros et al., 2014). It is similar to the principle behind an artificial mechanism called contrastive Excitation Backprop (c-EB)—a top-down excitation mask increments attention to the target features, whereas an inhibitory mask decrements attention to distractors (Zhang et al., 2018). Zou et al. (2020) modified the c-EB network to use in an action-based goal-driven perception task conducted with a Toyota HSR. The system each time guessed an action based on the updated neuromodulatory head (see section 4 for more details). Based on different camera views of a scene, the neural network increased attention to the objects related to a guessed action and decreased attention to distractors (see Figure 6). Selecting the highest attention region further helped with object localization and prediction. The top-down model, which combined ideas from neuroscience with goal-driven perception in artificial networks, has been demonstrated by the HSR experiment to extract features and flexibly shift attention to intended goals. It could be modified to deal with more complex and changing AI scenarios in face of both familiar and novel goals.

Neurorobotic experiments by Gigliotta et al. (2017) showed that an artificial neural network (ANN) simulating the ventral and dorsal attentional networks (VAN and DAN) in the brain reflected the human pseudoneglect behavior in visual search. Pseudoneglect is a human bias toward starting to search with left-sided items, which was experimentally confirmed by Gigliotta et al. (2017) with a human experiment. The human experiment had participants select targets on a touch-screen tablet to cancel them. All participants searched for and canceled the targets starting on the left of the screen, indicating pseudoneglect. To mirror the experiment using a neurorobot, an ANN controlled a simulated robotic eye with an artificial retina and four degrees of freedom and an artificial hand to repeat the human experiment task. The ANN was evolved using a genetic algorithm to simulate the functions of the VAN, DAN, and interhemispheric connections in the human brain. Five populations of the ANN composed of 40 individuals performed the same task as the humans. Each population had a different neurocontroller with different connection constraints. The five neurocontrollers A-E were constrained as follows: (A) no constraints; (B) VAN to DAN pathways were excitatory during training; (C) Same as B but additionally retina to VAN connections were excitatory; D) The left hemisphere received information only from the right, contralateral visual hemifield and there were no inhibitory connections between the DAN in the right hemisphere and left hemisphere; (E) Same as D, but visuo-attentional connections were constrained to be excitatory. The first canceled target during each of the experiments tended to be in the center for population A, to the left of center for populations B and C, and to the right of center for populations D and E. Therefore populations B and C showed a leftward bias similar to pseudoneglect found in the human experiment, with results showing that population C in particular most closely matched human performance among the populations. When comparing all the targets canceled between humans and the ANNs, the order of cancellations of population C closely matched humans. Therefore, the neurorobotic study reinforced evidence that the reason for pseudoneglect in human visual search are the result of hemispheric asymmetries between VAN and DAN with a general excititory influence of VAN on the ipsilateral DAN. The study gives evidence that ANNs can exhibit behavior similar to humans when embodied in a neurorobot and be used to help explain human behavior and functions of the brain.

## 6. Locomotion

Neurobotics has been used to study locomotion over land, air, and water (Lock et al., 2013; Ijspeert, 2014). Animals have evolved to consume low energy for periodic and passive movements with their muscles but demonstrate incredible agility over a range of dynamic environments (Dickinson et al., 2000; Alexander, 2003; Biewener and Patek, 2018). Therefore, biologically-inspired models of locomotion would allow robots to develop better sensorimotor skills and accomplish complex tasks (e.g., delivery, predator-prey, search, and rescue, etc.) in the real world (Zabala et al., 2012; Hwu et al., 2017; Nelson et al., 2018; Krichmar et al., 2019). Because their actions are repeatable and their control systems are reprogrammable and durable, neurorobots can be reverse-engineered to better understand the actual interactions among an animal's body, control mechanism, and living environment (Ijspeert, 2008, 2014; Goulding, 2009; Kiehn, 2016).

**Figure 10:**
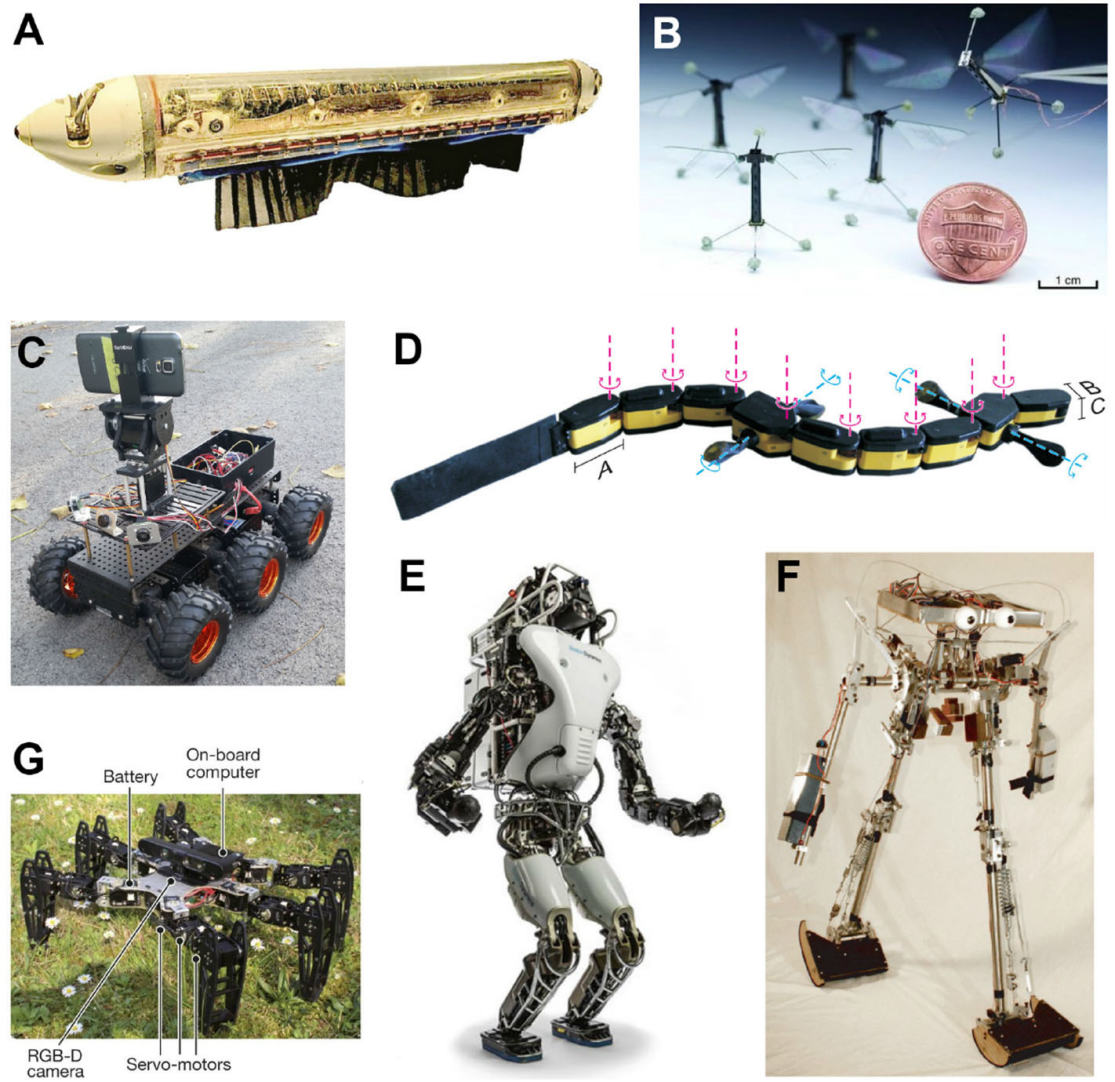
Examples of neurorobots that feature different locomotion control mechanisms. **(A)** A knifefish-like robot with a ventral ribbon fin, which allows for inward-traveling waves (Sefati et al., 2013). **(B)** A flapping-wing robotic fly with a pair of independently actuated wings, which allows for exert control torques about all three body axes (Ma et al., 2013). **(C)** The 6-wheel-drive Android-Based Robotics Platform, controlled by an Android smartphone and ideal for an outdoor neuromorphic system of navigation (Hwu et al., 2017). **(D)** Salamandra robotica II, an amphibious robot that alternates between swimming and walking, according to the strengths of stimulation on the central pattern generator (CPG) network (Crespi et al., 2013). **(E)** Atlas-Unplugged, Boston Dynamics' first untethered biped robot, which requires joint control with high-torque actuators at all times for perception, mobility, and manipulation in simulated disaster scenario (Nelson et al., 2018). **(F)** The Cornell biped, a passive-dynamics-based robot which features efficient and human-like gait (Collins and Ruina, 2005). **(G)** A hexapod robot to test locomotion adaptation after getting injured with damaged, broken, and missing legs (Cully et al., 2015).

A fish-like robot could regulate the amount of force on its deformable fins to alter the surrounding water flow and swim freely (see Figure 10A) (Lauder et al., 2007; Long, 2007; Sefati et al., 2013; Porez et al., 2014). With similar fluid dynamics in air, a flapping-wing robot could rotate and flap its actuated wings with force sensors to generate necessary lift and modulate the direction for flight (see Figure 10B) (Dickinson et al., 1999; Ma et al., 2013; Elzinga et al., 2014). A ground robot has either continuous contact with the ground through its wheels or tracks (see Figure 10C) or discrete contact through its legs – the latter allows more convenient travel on uneven terrains with limited footholds but usually requires a more complex control system for body-limb coordination (Saranli et al., 2001; Raibert et al., 2008; Hamed et al., 2019). For example, Salamandra robotica II (see Figure 10D), a salamander robot that can swim and walk, is driven by the central pattern generator (CPG) network distributed along the entire spinal cord and four limbs. A single chain of amplitude-controlled phase oscillators with bilateral coupling is implemented for the body, whereas a single oscillator is placed for each limb and locally connects to the corresponding oscillator on the spine (Crespi et al., 2013). The body CPG has the natural tendency to produce traveling waves under the activation of a tonic drive. At high stimulation, the limb CPG saturates and stops oscillating for swimming-like patterns. At low stimulation, the strong couplings from limb to body oscillators “override” the natural traveling waves and instead force production of standing waves for walking-like patterns (Ijspeert et al., 2007). Another group of multi-legged robots is the humanoid biped robot, which could be further distinguished into two categories of control. The first category [e.g., Asimo from Honda (Sakagami et al., 2002) and Atlas from Boston Dynamics as shown in Figure 10E (Nelson et al., 2018)] features versatile joint control with high-torque actuators at all times but sacrifices energy efficiency (Ijspeert, 2014). The second category [e.g., the Cornell, Delft, and MIT bipeds (Collins et al., 2005)] instead applies human-like free-swinging motions that reflect the passive dynamical properties of the musculoskeletal system (see Figure 10F for the Cornell biped) (McGeer, 1990; Collins et al., 2001; Collins and Ruina, 2005). All these various types of neurorobots were designed to possess sensorimotor skills that match a well-thought-out level of abstraction based on biological features for different spatial circumstances.

Neurorobotics has been used to investigate adaptive locomotion in response to perturbations, such as limb injuries or terrain variations. In locomotion experiments with a hexapod robot (see Figure 10G), a neural network with prior knowledge of potential behaviors and their values was used to rapidly adapt the robot's gait in response to a broken or altered leg (Cully et al., 2015). In this approach, the robot mentally simulated different gaits, and then chose the new gait with the best chance of recovering from the perturbation. In another approach, decentralized control on the multi-segmented body of a legged robot maintained walking-like pattern at the local segment level based on the mechano-sensory feedback (i.e., with leg contacting the ground) alone when the severed spinal cord blocks the descending (brain) control (Suzuki et al., 2019; Yasui et al., 2019). Such sensitive adaptability enables these neurorobots to still perform robustly in the face of the damage or environmental interference, which could in turn improve the understanding of animals' compensatory behaviors and even reduce the needs of some fragile animal experiments.

## 7. Neuromorphic Robots

Neuromorphic engineering is the design of integrated circuits inspired by the energy-saving form and function of neurons (Indiveri et al., 2011). Compared to traditional computer chip designs, neuromorphic chips consist of small connected units running asynchronously and in parallel with intermittent spiking activity. This results in hardware that can compute with magnitudes less energy, which is particularly useful for applications requiring energy conservation, such as robotics. Neuromorphic algorithms situated in robotic platforms have been able to explain how environmental constraints such as size and power can shape the cognitive and neural mechanisms of living agents.

**Figure 11:**
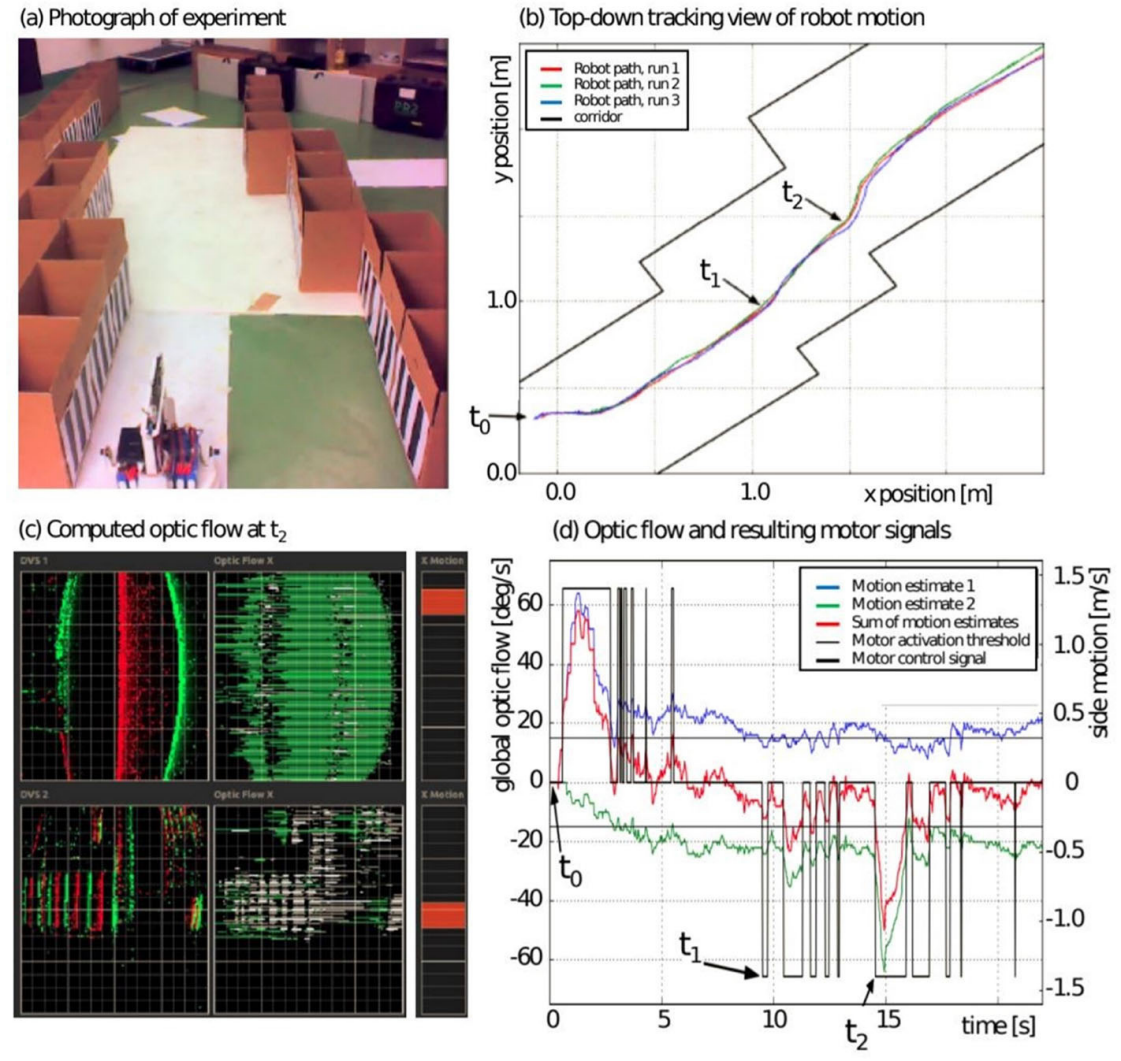
Trajectory stabilization using optical flow. **(A)** Photograph of experiment arena, front end of the mobile robot. **(B)** Top-down tracking view of arena and robot path in three consecutive experiments. **(C)** Left: display of observed events from eDVS, middle: derived horizontal optic flow (green and white indicate different polarities). Right: combined flow estimate equal to lateral motion motor command. **(D)** Time series of one experiment showing global optic flow (blue, green, and combined in red) and resulting motor commands. Figure and caption are reproduced from Galluppi et al. (2014).

Because neuromorphic hardware has low size, weight and power using event-driven, massively parallel, and distributed processing of information, it is ideal for autonomous navigation settings in which the mobile platform has a limited power supply and limited connectivity (Hwu et al., 2017). Autonomous mobile platforms have been developed to take advantage of these properties. For example, Galluppi et al. (2014) used the SpiNNaker (Painkras et al., 2013) neuromorphic processor on a mobile robot and an embedded dynamic vision sensor (eDVS) for spiking visual input. The robot demonstrated trajectory stabilization using Optic Flow (OF) using an experimental setup that emulates flight experiments performed with bees (see Figure 11). This suggested that a cognitive behavior, such as rapidly moving through cluttered spaces, could be realized in low power systems with spike-based calculations.

For path planning, a spiking wavefront propagation algorithm was created, which was compatible with neuromorphic hardware (Hwu et al., 2017). Spiking neurons were connected in a topographical map corresponding to locations in 2D space. An efficient path between the start and goal location was obtained by examining the spike times of neurons and determining which sequence of spikes arrived at their destination first. The algorithm was run on an Android phone, which was mounted on a robotic platform. The robot planned paths through different terrains and altered its routes based on the cost of traversal across its environment. The algorithm showed how the timing of spikes and the varying axonal delays between neurons could be used to plan efficient paths and adapt to environmental change. Using the same Android based platform, a computer vision road following algorithm was developed for the TrueNorth with visual input being taken from the camera of an Android phone mounted on the robot and a self-driving convolutional neural network, which ran on TrueNorth (Hwu et al., 2017). Video frames from the phone were transformed into spiking input and sent to the TrueNorth for processing. Output spikes from the TrueNorth were sent back to the phone to determine whether to steer the robot left, right, or forward. The robot was able to autonomously follow a steep mountain road shown. Hwu et al. (2017) demonstrated that when the computation for path planning and navigation algorithms were offloaded to energy-efficient neuromorphic hardware, the robot was able to explore unknown territory in real-world outdoor environments for extended periods of time.

A cluster of nine TrueNorth neuromorphic processors was used by Andreopoulos et al. (2018) to implement a low power and high throughput stereo vision system. The end-to-end neuromorphic system comprised of two DAVIS cameras (Brandli et al., 2014) that captured spiking visual input at 240 × 180 spatial resolution and microsecond temporal resolution, which were then routed directly to the neuromorphic cluster for stereo disparity computation. Their proposed spiking stereo disparity algorithm performed a series of operations using the spiking neurons of TrueNorth, namely rectification, hierarchical spatiotemporal scaling, noise removal, epipolar region proposal, stereo matching, and winner-take-all, to output disparity maps at 400 frames-per second using less than one watt of power. Its high throughput enabled depth estimation and 3D reconstruction of fast moving objects, such as rotating fan blades, which was not possible in case of traditional frame based camera input. Moreover, the stereo matching algorithm achieved accuracy from high temporal resolution and hierarchical processing of input spikes. This TrueNorth stereo vision system demonstrated that retina inspired spiking vision sensors and brain inspired neuromorphic processors enabled real time and accurate disparity calculation through high temporal resolution data capture and processing, while consuming fraction of the power budget of traditional computers.

Neuromorphic robotics experiments have also been used to explain agent interactions, such as predator and prey relationships (Moeys et al., 2016). In this study, two robots were used with one behaving as the predator and the other as the prey. Both robots had a laser scanner to detect and avoid collisions, but the predator robot additionally had a Dynamic Active Pixel Vision Sensor (DAVIS), a neuromorphic sensor that takes visual input both as event-based frames and standard image frames. To avoid the predator, the prey robot followed a semi-random policy using its laser scanner to avoid obstacles and collisions with walls. The prey robot did not learn to actively avoid the predator but used the policy to autonomously move without colliding into walls or the predator. The predator robot learned to actively follow the prey robot using vision as input to a trained CNN artificial neural network. The goal behavior of the predator robot was to keep the prey robot within the center of its field of view and move toward the prey robot to a certain safe distance to catch the prey while avoiding collision, and search for the prey robot if it was not in the center of its field of view. If the laser sensor on the predator robot detects an imminent collision while the prey robot is in the center of the predator's view then the prey is considered caught. Despite neural networks being thought of as black boxes, visualizations of the visual processing of the CNN can help explain the neural network functions as well as the behavior of the robot. Similarly the behavior of the robot can help validate the network performance and be used to improve the performance of the network. The predator robot experiments give an example of how artificial neural networks can be combined with neuromorphic hardware to drive biologically inspired behavior.

**Figure 12:**
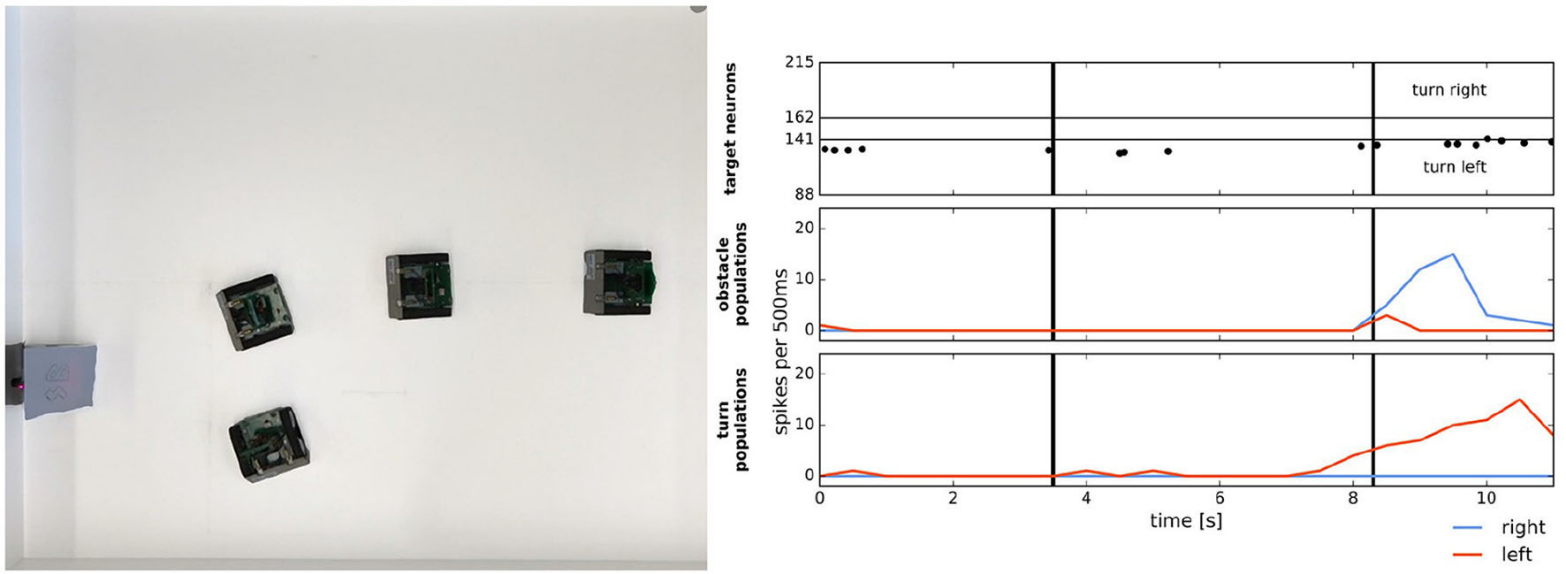
Simple target acquisition: single stationary target. **Left**: Overlay of video frames from the overhead camera. The robot approaches a stationary target on the left-hand side of the arena from right to left. The robot turns left toward the target until it perceives it as an obstacle and makes an obstacle avoidance maneuver. **Right**: Time-course of the spiking activity (raster plot) of the target-representing (WTA) neurons on the ROLLS chip (top plot) and summed (over 500 ms and over populations) activity of neurons in obstacle representing and drive populations on the ROLLS chip. Vertical lines mark time points that correspond to two middle positions of the navigating robot. Figure and caption are reproduced from Milde et al. (2017).

In Milde et al. (2017), a mixed-signal analog-digital Reconfigurable On-Line Learning (ROLLS) neuromorphic processor was interfaced with a Dynamic Vision Sensor (DVS) robotic vehicle and developed autonomous neurally inspired obstacle avoidance and target acquisition behaviors. The neural architecture used neural populations to determine the steering direction and speed of the robot based on the event-driven DVS. When enough of these events triggered a population to fire in either the left or right field of view, then an object was detected and the output of the neural network caused the robot to move in the opposite direction of the detected object to avoid it. The performance of the system was verified with over 100 runs in different settings, which included avoiding one or more static obstacles, avoiding moving obstacles, obstacle avoidance in a real-world office, and target acquisition of a blinking LED. Figure 12 shows both the target acquisition behavior and obstacle avoidance of the robot in action. The tracking and avoidance observed in the robot illustrated how such behavior could be realized with energy-efficient neuromorphic computation using sparse spiking activity. The ROLLS experiments demonstrate the value of the greater computational efficiency of using a mixed-signal neuromorphic system. Using analog sensory signals directly for low-level processing one can build complex neural architectures to solve cognitive tasks such as task planning and map building with fewer resources than conventional digital implementations.

Living animals learn by exploring the environment. Chen et al. (2019) demonstrated such automatic behavior learning in a neurorobot with the neuromorphic snake-like robot NeuroSnake. The NeuroSnake used a miniature embedded Dynamic Vision Sensor (meDVS) for sensing and the SpiNNaker infrastructure for neuromorphic computing capabilities. A CPG network was used for snake-like autonomous locomotion. Experiments with the NeuroSnake involved learning two behaviors automatically that were further integrated into a complex autonomous pole climbing task. Animals can learn a new behavior by randomly performing the right behavior and remembering sensory data from the performance, so the NeuroSnake experiments were designed to mimic this. The first automatically learned behavior was automatic turning. In the experiment the NeuroSnake had to turn toward an LED by scanning with its head and slithering toward it to record the LED detection with the meDVS once the LED is in the center of the meDVS view. With the sensory and motor values the NeuroSnake learned the turning motion automatically using a neural network implemented in Nengo and the Neural Engineering Framework (NEF). The second automatically learned behavior was adapting the slithering gait to the environment. To accomplish this the robot was placed in the center of two LEDs attached to two poles at a fixed distance in the slithering direction. The robot detected the positions of the LEDs and then performed a slithering gait with a random amplitude, recording the sensory state and motor values only when the robot passed through the two poles. After the neural network learned the new rule, the robot was able to adapt the slithering gate to novel situations such as passing through a narrow space. The two behaviors were then integrated to learn autonomous pole climbing. The success of the robot in automatically learning the behaviors explains the neural network functions similarly to how real world animals automatically learn new behaviors. The neurons in the network learn to approximate the functions to perform the behaviors autonomously, meaning that by simply giving a robot examples of a desired behavior the robot can automatically develop learning rules instead of needing the rules explicitly defined.

Fischl et al. (2019) showed that advances in neuromorphic computing provide the hardware solutions for complex neural models needed to produce socio-emotional robots. They developed a robot that used a simplified primate amygdala neural network model to determine an emotional state from visual input that would elicit a behavioral response. The behavioral responses the robot could exhibit were happy, distressed, and neutral. The robot was able to successfully interact in real time with a diverse group of people, accurately detecting a person's facial expression and computing the appropriate emotional response, happy for smiling, distressed for frowning, and neutral for a neutral expression. The robot drove toward smiling people, away from frowning people, and remained stationary for those with a neutral expression. Additionally, the amygdala neural network activity was analyzed to explain and validate that the functioning of the behavior was the result of the network mirroring the functions of the biological amygdala. The robot differed from many existing robots because it computed an internal emotional model based on socially-relevant visual inputs to determine the robot's emotional state using a distributed processing system that could be used for longer term, more complex emotional modeling.

In general, these neuromorphic robots demonstrated how neural models can be made efficient enough to operate in real environments without external power sources or connections to cloud servers. They shed light on how biological organisms might achieve these feats with extremely energy-efficient nervous systems.

## 8. Social Interaction

A common aim in robotics and artificial intelligence is to aid humans in their daily tasks. A robot's ability to connect and communicate with other agents, human or mechanical, requires the ability to recognize and express thoughts and emotions. The manner in which humans and other animals interact varies, from gestures and facial emotions to spoken and written language. Modes of communication also change during brain development. Neurorobotics helps to explain models of social interaction, with the ability to directly compare with human behavior and directly interact with the humans as well.

### 8.1. Affective Cognition

**Figure 13:**
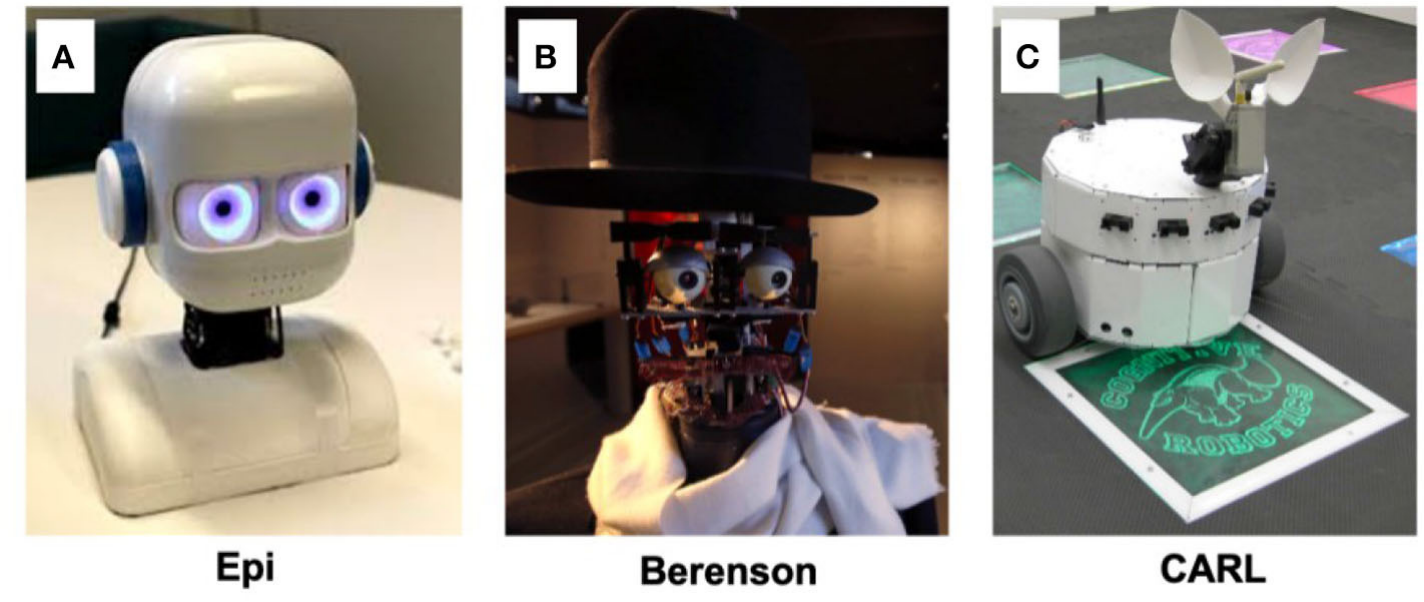
Examples of neurorobotics increasing AI understandability through interaction with people. **(A)** *Epi*, a humanoid child-like robot with the ability to change iris color and pupil size (Johansson et al., 2020). **(B)** *Berenson*, a humanoid robot capable of making facial expressions in line with its emotions regarding different art pieces (Pereira, 2016). **(C)** *CARL*, an anteater robot reacting with fear and excitement at colored stimuli (Cox and Krichmar, 2009).

The traits that define and set humans apart from machines are unsurprisingly the most difficult to emulate and explain in artificial intelligence. Many neurorobotics studies endeavor to model the cognitive processing of emotion and its physical expression. For instance, Balkenius et al. (2019) study the connection between the emotion of arousal and levels of noradrenaline with the brain. They note that implementing arousal in a robot allows them to process and react to environmental change, affecting decision making and choosing between explorative and exploitative behaviors. The physical display of arousal works as a social cue, affecting interactions with other agents. Through a special eye design involving circles of overlapping blades as irises (Johansson et al., 2020), they showed that a robot can display mental state through stages of alertness and arousal during problem solving and decision making (Figure 13A). An image of the robot is seen in Figure 13A. Neurorobots equipped with more facial actuators, as in Figure 13B, can express a larger range of emotions, such as sadness and happiness. In one instance, a robot controlled by a cognitive model for imitation learning was able to learn how to produce facial expressions by observing human faces (Boucenna et al., 2014). Maintaining a tight control loop between expression of affect and physical responses promotes a nearly instinctual understanding between artificial and human agents. The benefit of adding affect to robots is an increased trust and understanding between humans and robots, a key component of explainability. As it is shown that humans react more positively to robots with positive expressions and negatively to negative expressions (Kirby et al., 2010), the study of affect has the ability to improve human robot interactions and explain them in a quantitative way.

### 8.2. Imitation Learning

Imitation learning, in which primates and other animals learn by observing others, may be a means toward teaching robots to make complicated movements. The mirror neuron system is believed to play an important role in this process. Mirror neurons are active not only when primates themselves execute actions such as grasping objects, but also when they watch another animal performing the same actions (Rizzolatti and Craighero, 2004). Experiments inspired by the mirror neuron system showed that robots can imitate the behaviors or movements from observation (Schaal, 1999; Billard and Matarić, 2001; Schaal et al., 2003; Tani et al., 2004). For example, Billard and Matarić (2001) designed a robotic system with a controller composed of a hierarchy of artificial neural networks. Each component of the neural network gave an abstraction of functionality of a brain region involved in motor control. By reading human arm movement data recorded by a video and marker-based tracking system, the robot was able to replicate the two-arm movements of humans.

**Figure 14:**
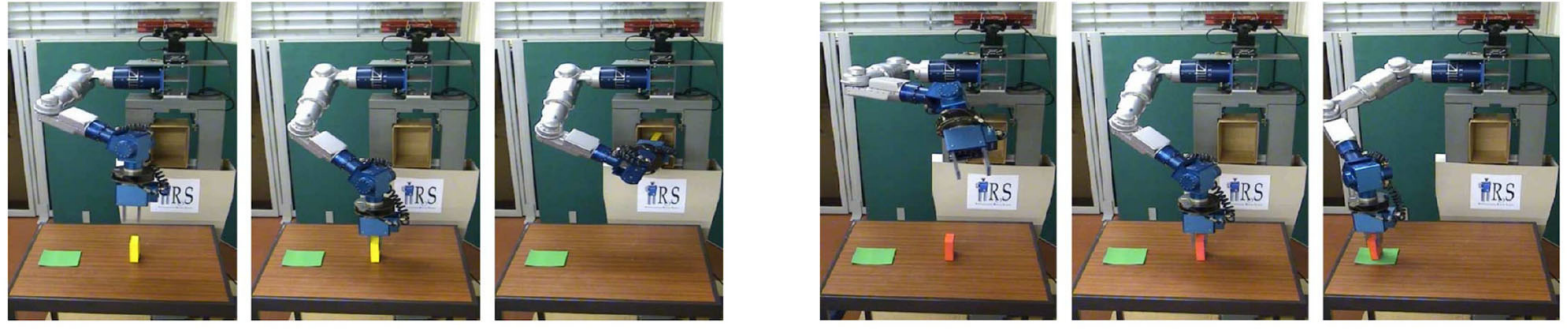
Examples of neurorobotic imitation learning tasks. **Left**: Three frames extracted from the sequence of “Grasping To Take” executed by the robot. “Taking” consists of placing the object into the box inside the robot. **Right**: Three frames extracted from the sequence of “Grasping To Place” executed by the robot. “Placing” means placing the object in a desired position. Figure and caption are reproduced from Chersi (2012).

The mirror neuron system is not only about copying another person's movements, but also may be involved in understanding another person's intentions (Iacoboni et al., 2005). Chersi (2012) implemented a spiking neuron model of the mirror system on a humanoid robot to study how the mirror neuron system might lead to understanding anothers' intention. The robot was instructed by the researchers to observe, learn and imitate based on a set of gesture commands. The experiment was divided into three phases of observation, learning and imitation. In the beginning of the experiment, the robot was in “observation” mode during which researchers set up the working area. Once the area was set up, the human demonstrator used a gesture sign to tell the robot to switch to “learning” mode and started to demonstrate action sequences to be learned. Two types of action sequences were demonstrated during this phase and each of them represented a distinct task. The task of “eating” included an action sequence of “reaching,” “grasping,” and “taking” that would take the “food” to a virtual “stomach” (a box in the experiment). To solve another task of “placing,” the robot needed to take another action sequence of “reaching,” “grasping,” and “placing” that would place the “food” to a desired position (see Figure 14).

In ‘the ‘imitation” mode, the robot first needed to understand which task to perform by analyzing the cues in the present scene and then replicating that action sequence to complete the task. The neurorobotic architecture implemented in this work performed well in both understanding the intention and replicating the action sequences. In these neurorobotic imitation experiments, the movement behavior executed by the robot helps to verify and explain the mechanism of mirror neuron system. For example, the successful imitation learning of the robot from Chersi (2012) supported the Chain Model which is the base of its framework and hypothesized that motor and mirror neurons in the parietal and premotor cortices are organized in chains encoding subsequent motor acts leading to specific goals. As a result, these experiments may lead to a beneficial system for learning movements.

### 8.3. Language

Language represents a high level of cognitive process. During verbal communications, our brain performs several complex processes at once, organizing thoughts into words and generating utterances, and also paying attention to the other agents' sentences and actions. More importantly, how languages were developed to map words to our internal representations of the external world is an intriguing question. However, it is difficult to create controlled experiments to observe how human beings learn a language. Neurorobots provide a great tool for studying how language emerges, as robots can be easily designed with no prior knowledge of lexical or syntactic representations. Neurorobots are also advantageous in that they present an embodiment of the neural processes and sensorimotor knowledge necessary to acquire and use language.

With a population of babbling robots, Oudeyer (2006) demonstrated that a phonetic system could emerge through agent-agent interactions. The robots had a vocal tract model that could produce vocalization through different activations in their neural motor maps. The robots also had an ear model that transformed acoustic signals to neural responses in its perceptual neural map. The two maps were connected to allow the agents to learn the auditory-motor mapping. Over time, the population of agents developed a shared system of vocalizations that resembled the vowel systems observed in human languages. This neurorobotics experiment allowed researchers to seek the origin of language development from the perspectives of social interaction and sensorimotor integration.

**Figure 15:**
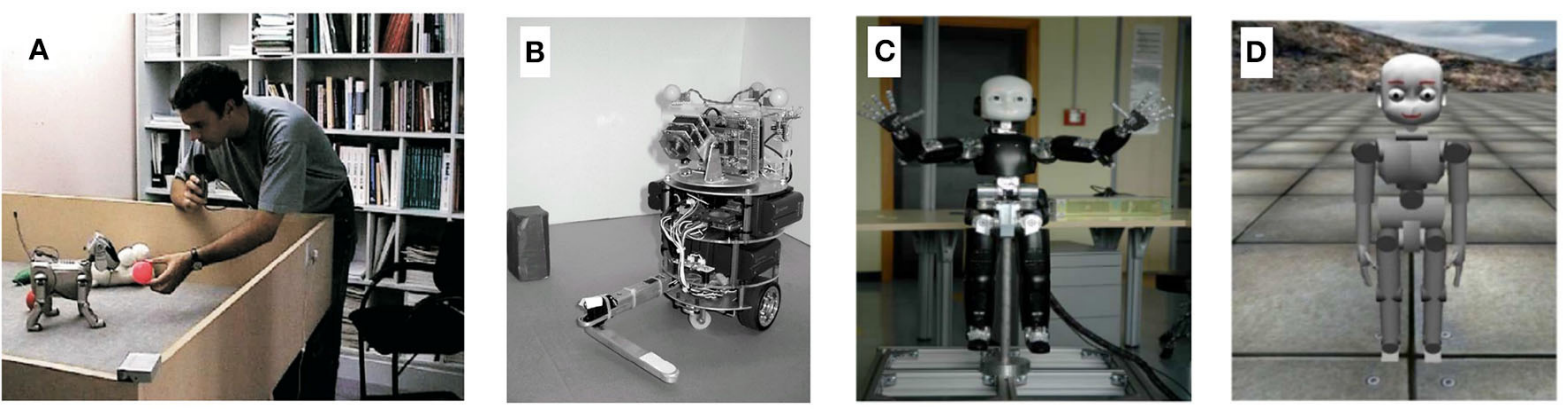
Neurorobot experiments demonstrating language acquisition and development in coordination with cognitive and motor controls. **(A)** A human mediator interacts with the AIBO robot using language games to teach it words (Steels and Kaplan, 2000). **(B)** A mobile robot used in Sugita and Tani (2005) which performs different behaviors such as pointing at, pushing, or hitting an object according to the instruction. **(C,D)** The iCub robot and its simulated version used in Tikhanoff et al. (2011) demonstrating multimodal language acquisition.

Understanding a language starts from acquiring an understanding of the semantic meaning of words. This involves learning the associations between words and objects or actions in the world, which can be achieved through: (1) individualistic learning where the agent receives input as paired examples of speech and specific situations, or (2) social learning, where a mediator and a learner is present (Steels and Kaplan, 2000). A mediator usually has more knowledge and takes the lead in a learning process by giving feedback to the learner. Steels and Kaplan (2000) used the AIBO robot to perform a series of experiments to examine the importance of social learning in the acquisition of word meanings. The experiments were composed of different language games in which the robot had intense interaction with a human mediator (Figure 15A). The robot stored the relation between object views and words in its associative memory, and the associations were learned through reinforcement learning (Sutton and Barto, 1998). The human mediator provided positive and negative verbal feedback that helped increase and decrease the association between the object and the word. These experiments showed that social learning facilitated word learning by constraining the situation and by providing reinforcing feedback, both of which helped the robot to gather good samples of object-views and words that shared clear causal inferences.

A popular theory in linguistics states that the meaning of a sentence can be directly inferred by combining the meaning of the words in it. This is known as the compositionality of language. Sugita and Tani (2005) applied a connectionist model on a mobile robot (Figure 15B) to illustrate how the compositionality of semantics of a simple language could be learned through the interaction between linguistic and behavioral processes. In their model, Recurrent Neural Networks (RNNs) were used to generate and recognize word sequences and sensory-motor sequences. During training, the network learned associations between sentences and behavioral sequences. During testing, the robot was required to generate correct behaviors corresponding to the given sentences, and the robot also showed the ability of generalizing linguistic knowledge to sentences not learned during the training phase. With no explicit knowledge of words and behavior routines specified before training, the robot was able to learn the semantics of a simple language, by representing the meaning of verbs and nouns independently, and assembling these meanings to understand sentences. The authors pointed out that the robot achieved the compositional semantics through iterative interactions between linguistic and behavioral structures, in which dynamical structures are self-organized.

Language is often used to initiate goal directed tasks. This process integrates perception and learning, which requires the agents to be able to understand instructions, and to be able to handle and manipulate objects in an adaptive manner. Tikhanoff et al. (2011) performed a series of robotic simulation experiments with the iCub platform to demonstrate how robots could integrate multiple neural networks processing vision and speech signals and learn names of objects and actions (Figures 15C,D). The robot's motor system had two neural network controllers: a feedforward network trained with backpropagation, which allowed for reaching toward objects, and an RNN, which is trained online to allow for grasping objects. The robot also received two kinds of sensory inputs: visual and speech inputs, which were processed with an visual segmentation algorithm and a real-time speech recognition system known as the CMU Sphinx system. The robot used a goal selection feedforward neural network to integrate various sensory processing capabilities and produces one of four actions: idle, reach, grasp, and drop. During training, an object and a speech signal was given to the robot. After training, the robot learned to expect a speech signal before it performed an action to the given object. With the given verbal instruction, the robot successfully performed a sequence of actions following the instruction. These experiments showed how integrating vision, action, and language modalities allowed an agent to form sensorimotor representations of the world and manipulating objects in the world.

## 9. Public Outreach

Explainable artificial intelligence plays a part in introducing technology to members of the public who may have little knowledge of computer science and neuroscience. Integrating new technology requires trust from its users, and trust develops from good communication and social interaction. Compared to attempts at explainable artificial intelligence from conventional machine learning methods, neurorobotics has the advantage of being able to communicate with humans as physical entities in the world. For instance, the Cognitive Anteater Robotics Laboratory at the University of Irvine, California, used a neurorobotics demonstration from a previous work (Cox and Krichmar, 2009), explaining neuromodulation concepts to lay audiences including young school-aged children (see Figure 13C). Complicated networks and algorithms became clear to explain as the concepts were related to universal human knowledge, such as emotions and personality traits. The colorful stimuli and animal likeness of the robot captivated viewers and fostered an interest in learning more about the implementation. Another example of public engagement was the display of robot Berenson at the Musée du Quai Branly during the exhibition Persona: Oddly Human + Emotion (Pereira, 2016). The robot, capable of expressing positive and negative facial emotions, was able to view works of art, determine negative and positive traits of the artwork, and reflect the affect of the works using facial muscles. Robots showing emotion are not only engaging, but also therapeutic. For instance, the CARBO robot processes tactile input, coaching children who have autism spectrum disorder on the relationships between touch, social interaction, and emotional response (Krichmar and Chou, 2018). The use of universal cues such as emotion, touch, and interaction are instrumental in creating understandable artificial intelligence.

## 10. Conclusion

In this paper, we reviewed a number of robotic experiments with varying devices and goals. The paper did cover much ground; ranging from sensorimotor to social interactions. However, the common theme is that the behavior of these robots helped to explain their neural control, and analysis of neural control made predictions on how neural activity can lead to behavior (see Figure 1). In order to understand intelligence, we sought to understand the full range of the inputs, outputs, and processing. We explored how inputs to the brain are processed through complex sensory perception systems, which are consolidated into concepts, contexts, and cognitive maps. We then explored how this information is utilized according to dynamic environmental changes and internal needs, through mechanisms of neuromodulation and attention. Next, we showed how neurorobotic demonstrations allow models to interact with the outside world through locomotion and social interaction, enabled by efficient neuromorphic designs. By interacting with the real world and reaching out to public audiences, neurorobotic demonstrations shows that explainability of biological and artificial intelligence comes from embodied interactions and engagement with the systems.

We have discussed a number of robot studies that provided neurally inspired solutions to artificial intelligence or that made predictions to inspire neuroscience experiments. Table 1 lists some of these key findings and the lessons learned. In many of these cases the lesson learned was a plausible mechanism to explain a behavior. These behaviors ranged from an orientation reflex in the case of the sound localization in the barn owl (Rucci et al., 1999), to a cognitive concept, such as forming memory schemas (Hwu et al., 2020). In both extremes, the interaction of the neural simulation with the robot's sensing and actuation leads to a possible explanation of how the brain and body interact to realize a behavioral outcome.

**Table 1:** Seminal examples covering the cross-section of neurorobotics, neuroethology, and explainable AI.

**Area**	**Lessons learned**	**Neuroscience** ** Prediction**	**Neuro** ** Inspired**
Perception	Reinforced AEC as a ubiquitous coding strategy. Used to optimize learning of smooth pursuit and vergence eye movements on iCub*^a^^,^^b^*	X	
	Suggested that fingertip kinematics can be adapted online for fine-grained tactile sensing in a closed-loop*^c^*		X
Memory and Navigation	Suggested hippocampal transition cells could lead to learning place sequences*^d^*	X	X
	Hippocampal SLAM system with SoA performance. Predicted entorhinal grid cells*^e^^,^^f^*		X
	Schemas memory due to interaction between mPFC and hippocampus. Contextual memory for robots*^g^*	X	
Neuromodulation	Neuromodulator interaction leads to tradeoffs between anxious and curious behavior *^h^*	X	
	Neuromodulators control goal adaptation and perceptions. Guesses user goals in human-robot interaction.*^i^*		X
Attention	Bottom-up saliency drives attention to locations quite different from their surround, similar to saccadic eye movements.*^j^^,^^k^*	X	
	Top-down attention focuses only on critical stimuli directed by goal-relevant inputs, similar to the cholinergic system.*^i^^,^^l^^,^^m^*	X	
	Pseudoneglect is caused by hemispheric asymmetries of the attention network.*^n^*	X	
Locomotion	Biologically-inspired sensorimotor skills enable robots to demonstrate agile locomotion over land, air, and water. *^o^*		X
	Development of compensatory behaviors after leg or nerve cord injury*^p^^,^^q^*		X
Neuromorphic	Showed neuromorphic hardware and sensors can process visual input highly accurately in real time with low power*^r^*		X
	Learning rules do not need to be predefined and can be learned autonomously through environment feedback*^s^*		X
Social Interaction	Supported the theory that motor and mirror neurons in the parietal and premotor cortices are organized in chains encoding subsequent motor acts leading to specific goals*^t^*	X	
	Integration of multiple neural networks that process vision, action and language leads to the formation of sensorimotor representations of the world *^u^*		X

a *Beira et al. (2006)*,

b *Teulière et al. (2015)*,

c *Bologna et al. (2013)*,

d *Cuperlier et al. (2007)*,

e *Milford et al. (2004)*,

f *Milford et al. (2010)*,

g *Hwu et al. (2020)*,

h *Krichmar (2013)*,

i *Zou et al. (2020)*,

j *Itti and Koch (2000)*,

k *Hoffman and Subramaniam (1995)*,

l *Oros et al. (2014)*,

m *Zhang et al. (2018)*,

n *Gigliotta et al. (2017)*,

o *Ijspeert (2014)*,

p *Cully et al. (2015)*,

q *Yasui et al. (2019)*,

r *Andreopoulos et al. (2018)*,

s *Chen et al. (2019)*,

t *Chersi (2012)*,

u *Tikhanoff et al. (2011)*.

The added explainability via neurorobotics and neuroethology extends to benefits in society as a whole. For instance, the public outreach component described in section 9 shows how neurorobotics engages the public audience for a better appreciation of neuroscience and AI, perhaps even inspiring some to pursue a career in the field. Furthermore, the use of more explainable AI via neurorobotics leads to improvements in other fields, such as medicine and education. For instance, a neurobiological understanding of perception and action could lead to more intuitive neuroprostheses (Nordin et al., 2017), and a better understanding of navigation, attention, and social interaction could help with the development of assistive robots or telepresence robots to improve mobility and accessibility (Tanaka et al., 2013).

In many ways, neurorobotics is similar to the field of neuroethology. A major difference is that in the case of neurorobotics, the researcher has full access to the brain controlling the agent's behavior. This includes every neuron's activity and every synaptic change throughout the lifetime of the agent. Moreover, the neurorobotic researcher can control elements of the artificial brain and body through specific ablations and manipulations, which would be difficult or impossible in natural organisms. This makes neurobotics a powerful tool for explaining the complex behavior of artificially intelligent agents.

As AI systems and neural networks get more and more complicated, we may want to take a step back to observe the behavior of the system rather than over-analyze the network dynamics. We are often posed with the question, what is intelligence? And invariably, the answer is “I don't know, but I know intelligence when I see it.” Neurorobotics affords the opportunity to see intelligent behavior. As these devices and our analyses become more sophisticated, we hope that these robots will become more intelligent, we will understand why they are intelligent, and we may better understand our own intelligence.

## Author Contributions

All authors listed have made a substantial, direct and intellectual contribution to the work, and approved it for publication.

## Conflict of Interest

The authors declare that the research was conducted in the absence of any commercial or financial relationships that could be construed as a potential conflict of interest.
